# Artificial Intelligence Powered End-to-End Agricultural Machinery: Intelligent Transformation of Design and Maintenance Systems

**DOI:** 10.3390/s26196029

**Published:** 2026-09-23

**Authors:** Shengnan Tang, Xuhua Chen, Yong Zhu

**Affiliations:** 1 School of Mechanical Engineering, Jiangsu University, Zhenjiang 212013, China; tangsn@ujs.edu.cn (S.T.); 2222503128@stmail.ujs.edu.cn (X.C.); 2 State Key Laboratory of Mechanical Transmission for Advanced Equipment, Chongqing University, Chongqing 400044, China; 3 National Research Center of Pumps, Jiangsu University, Zhenjiang 212013, China

**Keywords:** artificial intelligence, intelligent perception, agricultural machinery automation, intelligent maintenance, precision agriculture, predictive maintenance

## Abstract

Reliable condition monitoring and fault diagnosis in agricultural machinery depend on interpreting sensor measurements under variable field conditions. Noise, occlusion, changing loads, and sensor degradation can obscure relevant features, while complementary measurements offer opportunities to reduce information gaps. This scoping review synthesizes 147 included evidence sources published from 2014 to early 2026, supplemented by 11 contextual publications. It examines sensing, feature extraction, and information fusion across mechanical modeling and design, perception, automation and control, and operation and maintenance. Modeling and design provide physical context for interpreting measurements, while perception and control connect state estimation with operational feedback. Applications in blockage detection and multi-component monitoring illustrate how complementary signals can support abnormal-state identification and operational intervention. However, improvements in perception accuracy or control performance do not independently establish improved fault diagnosis. Reported results often depend on local datasets or controlled platforms, and calibration errors, temporal misalignment, and changing operating conditions can compromise fused estimates. Evidence for reliable early warning, precise fault localization, and diagnostic robustness under degraded sensing remains limited. Future evaluations should compare fused and individual-sensor inputs under matched conditions, including noise, partial sensor failure, and changes across machines and seasons. Linking these comparisons to false alarms, missed faults, warning lead time, and maintenance decisions is essential for establishing the practical contribution of sensor fusion.

## 1. Introduction

Agricultural production is currently facing dual pressures from both the demand and supply sides. The continuous growth of the global population and the advancement of urbanization have led to a steady increase in food demand; meanwhile, the cumulative effects of rural labor shortages, scarcity of arable land resources, and climate change have made maintaining existing production capacity increasingly difficult [1]. The dominant production mode, which relies on manual experience, lacks accurate judgment of real-time field status in key stages such as irrigation, fertilization, and disease control. This makes it challenging to maintain stable operation quality across different plots and seasons. The resulting excessive resource input and yield fluctuations are the direct drivers promoting agricultural transformation [2]. Introducing technical means for acquiring real-time field information and adjusting operational parameters has become a practical necessity to improve agricultural production efficiency and resource utilization. Individual sensors can be affected by noise, occlusion, and changing operating conditions. Multi-sensor information fusion can reduce information blind spots and support robust state estimation, early fault warning, and fault localization.

In recent years, artificial intelligence (AI) related technologies in agricultural machinery (AM) have achieved substantial progress. In environmental perception, sensor-based machine-learning methods have supported crop-target detection, autonomous navigation, and precision field operations across multiple scenarios [3]. Furthermore, a recent systematic review has established machine learning and computer vision as core enablers of precision agriculture [4]. Regarding motion control, deep reinforcement learning has been applied to motion planning for multi-joint picking robotic arms, and operation coverage optimization for plant protection unmanned aerial vehicles (UAVs), achieving quantifiable improvements in tracking accuracy and spray coverage [5]. At the machinery-system level, AI has also been applied to machine-state estimation, fault pre-diagnosis, and agricultural Internet of Things data processing, linking field measurements with operational decisions [6].

However, most of these advancements remain limited to independent technology validation under controlled scenarios, and real field deployment still faces multi-level obstacles. Under actual farming conditions characterized by high vibration, extreme temperatures, and fluctuating humidity, electric AM requires more robust coordination between electric drives and overall vehicle control [7]. Some obstacles are primarily module-specific. The computational overload of model predictive control (MPC), neural networks, and multimodal perception reflects a direct conflict between algorithmic complexity and the power, thermal, and computing limits of field edge devices [8]. Non-destructive end-effector operation is more fundamentally constrained by mechanical design, crop biomechanics, and kinematic precision [9]. Meanwhile, field variability arises from spatial heterogeneity, temporal changes, semantic ambiguity, physical interaction complexity, and sensor signal degradation (Table 1). These disturbances can affect sensing modalities differently, making information fusion relevant to state estimation, early fault warning, and fault localization under field conditions. System integration becomes decisive when these module-specific constraints interact. At this integrated level, the failure rate of actuators for tractors and harvesters increases under muddy or sloped conditions, yet existing multi-actuator fault tolerance and human–machine collaborative safety boundaries remain imperfect [10]. Accordingly, this review distinguishes hardware-limited, environment-induced, mechanical-design, and system-integration barriers, while examining how sensing and information fusion connect dynamic modeling, perception, motion control, and operation and maintenance (O&M).

The retrieved records were subsequently screened through a three-step workflow. First, duplicates and records outside the specified timeframe were removed. Second, titles and abstracts were evaluated to exclude studies that did not focus on AM or treated agriculture merely as a generic context for data analysis. Third, full texts were assessed for relevance to at least one of the four core technical dimensions of intelligent AM (Figure 1): mechanical design, modeling, and manufacturing (Section 4); field environment perception and information acquisition (Section 5); autonomous navigation and motion control (Section 6); and equipment operations, maintenance (O&M), and fault management (Section 7). To be included, studies were required to demonstrate a clear connection to machinery hardware, field operations, embedded deployment, or machine-system decision-making. Conversely, exclusion criteria comprised purely agronomic studies without machinery involvement, generic AI algorithm papers lacking agricultural equipment validation, and non-peer-reviewed items unless they provided essential technical context.

While recent reviews have summarized agricultural automation, machine vision, or intelligent maintenance as relatively independent domains, this separation can obscure how sensor reliability and information fusion shape whole-machine performance. To bridge this gap, this review adopts an end-to-end organizing principle that connects machine design, sensing, information fusion, control, and operation and maintenance. Accordingly, the end-to-end perspective adopted here follows the information and control pathway from machine design and field sensing, through state interpretation, planning, control, and actuation, to operational feedback and maintenance, while recognizing that most available studies address only one or a subset of these stages. With particular attention to sensor reliability and information fusion, this review asks where module-level advances fail to produce robust, deployable machine-level intelligence. It also identifies the evidence needed to establish early fault warning, precise fault localization, and reliable operation under degraded sensing.

## 2. Materials and Methods

### 2.1. Review Design

This scoping review mapped AI applications in AM across four technical domains: dynamic modeling and mechanical-system design, intelligent perception, automation and control, and intelligent O&M. Within this evidence base, the narrative synthesis examines how sensor information, feature extraction, and information fusion support machinery perception, operational monitoring, and fault diagnosis. Modeling and design provide physical context, while perception and control describe the acquisition, interpretation, and operational use of information. The review distinguishes these supporting contributions from direct evidence of diagnostic performance. Reporting followed the Preferred Reporting Items for Systematic Reviews and Meta-Analyses extension for Scoping Reviews (PRISMA-ScR) [11]. The PRISMA 2020 flow-diagram template was used to report study selection [12].

### 2.2. Eligibility Criteria

Eligibility was structured using the Population-Concept-Context (PCC) framework. The population comprised AM, agricultural robots, unmanned vehicles, and machinery subsystems; the concept was AI contributing to design or modeling, perception, automation or control, operation, condition monitoring, fault diagnosis, or maintenance; and the context was agricultural production, including laboratory, simulation, controlled-field, and real-field settings. Eligible sources included peer-reviewed journal articles, review articles, conference papers, proceedings papers, and book chapters in peer-reviewed conference-proceedings volumes, all published in English or Chinese. The publication window covered 2014 through 30 January 2026. Publications assigned a later issue date were eligible only if an Early Access version had been indexed in Web of Science by 30 January 2026. During manual screening, priority was given to papers in journals classified in Categories 1 or 2 of the 2025 Chinese Academy of Sciences journal partition system; this prioritization guided screening effort but was not used as a standalone eligibility criterion.

Studies were included when AI methods contributed to the design, modeling, perception, automation, control, operation, condition monitoring, fault diagnosis, or maintenance of AM and addressed at least one of the four predefined technical domains. Non-peer-reviewed and retracted publications were excluded, as were studies in which AI was not directly applied to AM or whose principal focus did not correspond to one of the four domains. Papers concerned only with crop monitoring, agronomic prediction, or general precision agriculture were ineligible unless the AI method was integrated into an agricultural machine or a machinery-related operation.

#### Scope of Machinery-Related Evidence and the End-to-End Perspective

In this review, AM intelligence is distinguished from broader smart-agriculture research by requiring a direct functional link between an AI method and a physical machine or a machinery-mediated operation. Studies were therefore considered machinery-related only when their outputs were used to configure, guide, control, monitor, or maintain an agricultural machine or its subsystem. UAV remote sensing, crop disease recognition, biomass estimation, or fruit-ripeness detection were included only when the resulting information was explicitly connected to a machinery action, such as route planning, variable-rate spraying, harvesting, picking, or other machine-executed operations. Studies that only characterized crops or field conditions without influencing machinery behavior were treated as broader smart-agriculture research and were excluded from the machinery-focused synthesis.

The end-to-end perspective describes the analytical framework of this review. It connects mechanical modeling and design with sensing, state interpretation, operational decisions, actuation, and monitoring or maintenance feedback. Within this framework, modeling helps interpret machine responses, perception extracts relevant information, and control links estimated states to operational actions. Condition monitoring and fault diagnosis address the interpretation of abnormal machine behavior and its implications for intervention. Individual studies may address only part of this chain. Their contribution is interpreted according to the task and outcomes evaluated, without assuming that each study implements multi-sensor fusion or validates an integrated diagnostic system.

### 2.3. Information Sources and Search Strategy

The Web of Science Core Collection was searched through the Advanced Search interface on 30 January 2026; no individual citation index within the Core Collection (e.g., SCI-EXPANDED or ESCI) was imposed as an additional restriction. No other bibliographic databases, trial registers, reference-list or citation searches, Google Scholar searches, or grey-literature sources were used. Two additional sources were identified through targeted searches of publisher platforms: one from the SPIE Digital Library and one conference-proceedings book chapter from Springer Nature Link. Both sources were assessed against the same eligibility criteria and included through other methods; neither was counted as a Web of Science record.

The search was conducted in the Topic field using the following strategy: TS = ((“agricultural machin” OR “farm machin*” OR tractor* OR harvester* OR “combine harvester*” OR robot* OR “agricultural robot*” OR “picking robot*” OR drone* OR UAV* OR “unmanned aerial vehicle*”) AND (“artificial intelligence” OR AI OR “machine learning” OR “deep learning” OR “computer vision” OR “neural network*” OR intelligent OR smart OR autonomous OR “precision agriculture”) AND (design* OR manufactur* OR application* OR operation* OR maintenance* OR “fault diagnosis” OR monitor*)). This primary strategy is reproduced exactly as executed.

The search retrieved 76,358 records before screening. Publication year, language, document type, technical relevance, and journal category were assessed manually during screening rather than incorporated into the Boolean search string. On 15 March 2026, a separate targeted Web of Science search was conducted to identify recent publications on intelligent O&M. Eleven publications from this supplementary search were used only to support contextual technical descriptions and were excluded from the PRISMA flow counts. The complete supplementary search strategy and the eleven contextual publications are reported in Supplementary Table S3.

### 2.4. Screening and Eligibility Assessment

Retrieved records were imported into Zotero for reference management and duplicate checking; no duplicates were identified. Records were then exported to Excel for sorting and screening. One reviewer screened all titles and abstracts, and the other two reviewers checked the screening decisions against the eligibility criteria. Full-text eligibility was assessed by the same reviewers against the same criteria. No artificial-intelligence-assisted or automated screening tool was used. Disagreements were resolved through discussion or, when necessary, consultation with the supervisor.

### 2.5. Data Charting

A structured charting form was used to record bibliographic information, machinery or application context, technical domain, and AI-related information for the 147 charted evidence sources, when explicitly reported in the bibliographic record or source text. Information that could not be verified was coded as not reported. No duplicate charting by independent reviewers was performed. The charted dataset for the 147 evidence sources and the corresponding field definitions are provided in Supplementary Table S1.

To contextualize quantitative performance claims, we prepared Supplementary Table S4 as a source-level evidence table. The table records machinery type, crop or scenario, sample or dataset size, validation setting, comparison baseline, hardware or computing platform, reported metrics, and explicitly reported cross-region or cross-season testing. Metric names, units, and validation conditions were retained as reported by the original sources. No values were pooled, normalized, transformed, or ranked across studies. “Not reported in source” indicates that the information was not explicitly stated or could not be verified, whereas “Not applicable (review study)” indicates that the source did not contain a single primary experiment.

### 2.6. Evidence Synthesis and Critical Appraisal

The included literature was synthesized narratively within the four predefined technical domains. Across these domains, the synthesis examined how sensor information is acquired, represented, combined, and used in machinery operation, condition monitoring, and fault diagnosis. Modeling and design studies provided physical context for interpreting machine responses, while perception and control studies addressed information extraction and its operational use. Measurement, perception, and control outcomes were distinguished from diagnostic outcomes. Where studies combined multiple sensing inputs, their contributions were interpreted in relation to the reported inputs, comparison baselines, and validation conditions. Improvements in task performance were not attributed to sensor fusion alone without supporting comparisons.

Quantitative meta-analysis was not performed because the studies differed substantially in machinery platforms, agricultural tasks, datasets, sensor configurations, AI methods, validation environments, and outcome measures. No effect estimates were calculated, transformed, or imputed. No formal critical appraisal, heterogeneity analysis, or sensitivity analysis was conducted; findings were therefore not weighted according to study quality.

### 2.7. Protocol and Registration

This scoping review was not registered and no review protocol was prepared or made publicly available; accordingly, no protocol amendments were applicable.

## 3. Results

### 3.1. Study Selection

A total of 76,358 records were identified through the Web of Science Core Collection, and no duplicate records were found. After title and abstract screening, 76,206 records were excluded, leaving 152 reports for full-text assessment. Seven reports were excluded after full-text assessment, with the primary reasons documented in Supplementary Table S2. Consequently, 145 evidence sources from the database pathway were included. Two additional evidence sources, identified through targeted searches of the SPIE Digital Library and Springer Nature Link, were also assessed and included through other methods. Thus, 154 reports were assessed for eligibility across the two identification routes, of which 147 charted evidence sources were included in the final evidence set. In addition, 11 contextual publications, listed separately in Supplementary Table S3, were used only for background descriptions and narrative interpretation. They were not part of the 147 charted evidence sources and were excluded from the PRISMA counts. Together with two reporting-guideline references, PRISMA-ScR and PRISMA 2020, the manuscript cites 160 references in total, comprising 147 charted evidence sources, 11 contextual publications, and 2 reporting-guideline references. The study-selection process is presented in Figure 2, while the characteristics and charted fields of the 147 charted evidence sources are provided in Supplementary Table S1.

### 3.2. Characteristics of the Evidence Base

The 147 charted sources were published between 2014 and 2026, with 69 publications (46.9%) issued in 2025 and 28 (19.0%) in 2024. The 147 charted evidence sources comprised 109 research articles, 36 review articles, one proceedings article, and one book chapter. Of the 147 charted evidence sources, 127 were assigned to one of the four technical domains: 69 addressed AM automation, 40 intelligent perception, 15 dynamic modeling and mechanical-system design, and three intelligent operation and maintenance. The remaining 20 charted sources were coded as cross-domain because they addressed review scope, challenges, or future perspectives. These 20 sources were included within the 147 charted sources and were not additional publications. These domain assignments describe the distribution of the broader evidence base; they do not classify sources by fusion strategy or diagnostic validation. The synthesis therefore distinguishes the technical context provided by each domain from direct evidence concerning the contribution of sensor fusion to monitoring or fault diagnosis. Detailed characteristics of the 147 charted evidence sources are reported in Supplementary Table S1. Source-level machinery, validation, hardware, baseline, and metric information is summarized in Supplementary Table S4. The table shows that detection accuracy, localization error, path deviation, computational time, energy savings, and fault-diagnosis accuracy were evaluated under substantially different combinations of machinery, crops or scenarios, datasets, hardware platforms, operating conditions, and validation protocols. Therefore, these values are presented as descriptive source-level evidence rather than as directly comparable estimates.

## 4. Dynamic Modeling and Mechanical System Design

### 4.1. Dynamic Modeling and Data-Driven Simulation

Dynamic modeling characterizes how agricultural machinery responds to changing terrain, crop properties, and operating loads. These factors affect vibration, component motion, and material transport even when the machine operates normally. Understanding their effects is therefore necessary when interpreting changes in measured signals. The central question is whether an observed change can be explained by operating conditions or requires further investigation as a possible abnormal state. Simplified physical models and static empirical relationships may inadequately represent this variability under field conditions.

This problem involves both system-level dynamics and local contact mechanisms. At the system level, vehicle motion, powertrain vibration, and coupled component loads determine the overall mechanical response. Dynamic optimization can reduce vibration and improve equipment durability [13]. At the local level, interactions among soil, crop organs, and mechanical components affect traction and crop damage. Surface-morphology feature maps have been used to predict multiple outputs under complex working conditions [14]. The two scales are connected through load transmission. Local interactions generate forces acting on the machine, while system motion changes the conditions experienced by individual components. This connection helps explain why the same component can exhibit different responses across operating conditions.

Simulation combined with data-driven methods supports the analysis of internal operating processes. For combine-harvester vibrating screens, discrete element method (DEM) simulation and neural network prediction have supported guide-trough angle optimization to improve material distribution [15]. In cleaning chambers, airflow influences material transport, impurity separation, and grain loss. YOLOv8 has been applied to images derived from computational fluid dynamics (CFD) simulations to automate regional segmentation and key-point detection [16]. In this application, feature extraction operates on representations of simulated flow fields and supports design analysis. Together, these studies provide examples of predicting or extracting process characteristics that would otherwise require more extensive numerical analysis.

For monitoring applications, a model would need to predict the expected response under specified operating conditions and relate that response to available measurements. Comparing predicted and measured responses could then help identify changes not explained by the modeled loads. Such differences would still require interpretation because model mismatch, measurement error, and mechanical abnormalities can produce similar discrepancies. The studies above primarily establish modeling and optimization capabilities; using their outputs for abnormal-state assessment requires additional validation against measured machine behavior.

Table 2 places these examples within a broader comparison of data-driven modeling and approaches that incorporate physical information. Physics-informed learning is considered here as a possible extension for constraining predictions under changing conditions. Its usefulness depends on the validity of the governing relationships and the balance between physical constraints and data fitting. Physics–data integration and multi-sensor fusion have distinct roles: the former incorporates physical knowledge into modeling, while the latter combines information from multiple sensors. Their potential combination should be assessed through the reliability of the resulting state estimates.

### 4.2. Mechanical System Design and Adaptive Component Optimization

Mechanical design determines how agricultural machinery interacts with terrain and crops, and how its components respond during operation. The following examples cover terrain adaptation, platform stabilization, crop interaction, and functional-component optimization. Internal airflow sensing is then considered as a direct example of measuring an operating variable within the machine.

Terrain-adaptive chassis must accommodate slopes and soil-dependent traction constraints [17]. Wheeled, tracked, and wheel-leg configurations offer different trade-offs among travel efficiency, traction, and obstacle negotiation [18]. Electrified driving systems provide additional flexibility in power delivery and speed regulation [19]. These differences define the operating context in which slip, vibration, and loading should be interpreted. Figure 3 illustrates the chassis configurations discussed here.

Platform stabilization addresses changes in machine attitude during slope operation. A three-layer hydraulic frame combined with real-time control corrected body tilt to within ±1.5° in 3.6 s under the reported conditions [20]. This example links a defined machine-state variable, body inclination, to corrective actuation. End-effector design addresses a different problem: maintaining secure contact while limiting crop damage [21]. Telescopic suction cups combined with multiple fingers have been developed to balance picking efficiency and gentle handling [22]. For cherry tomatoes, flexible fingers using a grip-and-rotate motion have been combined with slip monitoring [23]. The latter provides a concrete example of observing the interaction state during manipulation and connects mechanical compliance with operational feedback.

Manufacturing methods provide a supporting design consideration. Additive manufacturing enables customized AM components, although dimensional accuracy and production cost constrain its application [24]. For working components, both feedback regulation and structural optimization can improve operational consistency. Contour-following wheel measurements combined with hydraulic compensation have been used to regulate cutter-bar height during harvesting [25]. Rigid–flexible coupling models and suspension optimization have also been used to suppress spray-boom oscillations [26]. These approaches act through different mechanisms. Height regulation uses measured position information to adjust operation, whereas suspension optimization changes the mechanical response to excitation. Distinguishing these mechanisms helps identify whether an observed improvement arises from feedback quality, controller action, or structural design.

Internal airflow sensing provides a more direct connection between measurement and machine-state assessment. Simulation can characterize cleaning-chamber airflow, but observing the operating machine requires a sensor suitable for the installation environment. A cylindrical anemometer incorporating pressure-sensing modules used deep learning to represent nonlinear measurement relationships [27]. The study evaluated airflow measurement performance and hardware cost, addressing the practical acquisition of an internal operating variable. This complements the simulation-based flow-field analysis discussed in Section 4.1.

Across these examples, mechanical design defines operating responses, feedback supports regulation, and internal sensing provides access to process variables. These contributions form a basis for interpreting machine behavior, while their validation remains specific to the reported design, control, or measurement task. Establishing diagnostic value requires determining whether the resulting information can distinguish normal operating variation from abnormal states. Section 5 examines the perception and information-fusion methods through which such measurements are represented and combined.

## 5. Intelligent Perception and Multi-Sensor Fusion in Agricultural Scenarios

### 5.1. Machine Vision-Based Object Detection and Identification

Machine vision extracts information about crop targets and operating conditions from visual measurements. Early approaches relied on handcrafted color and morphological features combined with conventional classifiers. RGB component features and a support vector machine (SVM) were used to distinguish citrus fruits from branches [28]. A framework combining a pulse-coupled neural network with an Elman neural network addressed overlapping and occluded apples [29]. Convolutional neural networks (CNNs) subsequently enabled learned feature representations, including those used for pepper detection [30]. These approaches illustrate different ways of extracting task-relevant information from images.

Subsequent developments address three distinct requirements: computational efficiency, feature discrimination, and access to complementary information. Lightweight YOLOv4 designs using depthwise separable convolutions reduce computational demands for deployment on field hardware [31]. Transformer and attention modules improve the representation of targets affected by occlusion or weak contrast, such as green fruits against foliage [32]. Multimodal perception and three-dimensional pose-estimation approaches extend the information available for target localization and action planning [33,34]. Their evaluation should identify the input modalities, the information contributed by each, and the requirements for alignment and calibration. Improvements from architectural changes should be distinguished from those obtained by introducing an additional sensing modality.

Perception metrics must also be interpreted according to the intended output. Figure 4 summarizes commonly used perception and control indicators and their operational implications. For operational-variable estimation, backpropagation neural networks have combined rice-ear pixel area, stalk height, moisture content, and grain-to-straw ratio to predict feed rate [35]. A YOLOv5s-based plant height–weight model has also been investigated for feed-rate estimation [36]. These studies use different representations and evaluation conditions, so their reported errors should not be treated as directly comparable. Modified DeepLabV3+ and YOLOv4 models have been applied to impurity and broken-grain monitoring in moving grain flows [37]. Lodge-Unet combines frequency-domain features with boundary-aware optimization for wheat-lodging segmentation [38]. Such feature combination within an image-processing model should be distinguished from integrating information across sensing modalities. Image and point-cloud representations, for example, provide appearance and spatial information that can complement one another [39].

The transition from object detection to operational-variable estimation changes the consequences of perception errors. Missing a fruit affects an individual picking action, whereas persistent bias in feed-rate estimation can influence speed regulation throughout an operation. Evaluations should therefore connect prediction errors with their duration and downstream effects, especially under dust, motion blur, and changing crop density. For multimodal methods, comparisons with individual inputs are needed to establish whether complementary information improves the relevant output under matched conditions. Figure 5 summarizes the relationships among the perception techniques and their AM applications.

### 5.2. UAV Remote Sensing and Multimodal Data Fusion

UAV remote sensing extends agricultural perception from local observations to spatially distributed measurements of crops and field conditions. Its contribution depends on how temporal information, sensing modalities, and environmental covariates are represented. Sequential models, including long short-term memory (LSTM) networks and gated recurrent units, capture changes across crop development and have been used for tobacco-yield prediction [40]. Hybrid CNN–LSTM models have combined multimodal data with temporal representations for prediction in the evaluated settings [41]. Temporal modeling and multimodal integration address different information gaps: repeated observations describe change over time, while complementary modalities characterize different properties of the observed scene.

Feature selection determines how much of the acquired information is useful for a particular task. Attention mechanisms incorporated into one-dimensional CNNs weight informative spectral bands and reduce the influence of redundant features [42]. Texture features derived from low-cost RGB imagery have also been combined with machine-learning models for maize above-ground biomass estimation [43]. Spectral weighting and texture extraction improve the use of available observations, but do not necessarily involve multiple sensors. They should therefore be distinguished from approaches that integrate independently acquired sensing modalities.

Environmental covariates provide another means of interpreting measurements under variable field conditions. Soil surface roughness has been incorporated into soil-salinity estimation to account for background effects in bare-soil observations [44]. Under vegetation cover, growth-stage-specific modeling and variables such as leaf area index help account for canopy interference [45]. Edge-based extraction of thermal canopy information has supported soil-moisture estimation in tea plantations [46], while crop-dependent classification has been used in salinity mappings [47]. These approaches illustrate how task-specific knowledge guides feature selection and interpretation when labeled data are limited [48]. Their transferability depends on whether the relationships between the selected variables and the target property remain stable across crops, growth stages, and locations.

Data-acquisition choices impose additional constraints on the information available for analysis. The non-dominated sorting genetic algorithm II (NSGA-II) has been used to optimize LiDAR flight paths and overlap while maintaining phenotypic extraction performance [49]. Thermal-imaging studies have examined flight altitude in relation to canopy-temperature measurements and physiological observations [50]. Spatial resolution has likewise been evaluated against processing requirements for wheat Fusarium head blight monitoring [51]. Meta-learning combined with particle swarm optimization (PSO) and transfer learning have addressed limited annotation in segmentation and crop–weed discrimination, respectively [52,53]. These developments support task-dependent sensing strategies in which acquisition and processing choices reflect the required output [54].

The main synthesis across these studies concerns the joint selection of measurements, features, and acquisition conditions. Additional modalities are useful when they contribute information that remains distinguishable and properly aligned within the combined representation. For machinery applications, spatial resolution and update frequency must also match the scale and timing of the intended operation. Plot-level yield or biomass estimation, for example, serves a different purpose from the local state information required for immediate machine adjustment. The reviewed studies provide evidence for agricultural estimation methods; their use in machinery decision loops requires a demonstrated connection between the estimated variable and the resulting action.

### 5.3. Perception Algorithms for Navigation-Relevant Scene Understanding

Navigation perception converts observations into representations of obstacles, crop boundaries, traversable space, and vehicle–terrain interaction. Unlike field-scale remote sensing, these outputs must remain relevant to the position and motion of the machine. The main technical progression is therefore from detecting individual objects toward describing the structure and operating context needed for navigation.

Lightweight visual models address the computational demands of this process. Modified YOLO backbones using partial convolutions and compact neck structures reduce model complexity for embedded detection [55]. Ghost modules and squeeze-and-excitation attention have been used to improve discrimination between obstacles and vegetation in the evaluated scenes [56]. Attention modules combined with bidirectional feature pyramids strengthen multiscale image representations [57], while compact MobileNetV4-based architectures support navigation-line extraction [58]. These methods primarily improve the processing of visual information. Their deployment should be assessed using acquisition-to-output latency and task-relevant detection errors, alongside model size and nominal inference speed.

Scene structure requires additional information beyond object labels. Enhanced DeepLabV3+ segmentation combined with B-spline fitting has been used to extract unharvested-area boundaries for combine harvesters [59]. Shared-encoder multitask networks jointly perform lane-line segmentation and obstacle detection [60]. Binocular vision combined with clustering and a bag-of-visual-words representation has also supported lodging-direction recognition [61]. These outputs describe geometry and spatial relationships needed for navigation. Their usefulness depends on whether boundaries remain continuous and correctly positioned near crop rows, field edges, and obstacles. Boundary errors in these locations can affect navigation even when average segmentation performance remains high.

Geometric and proprioceptive measurements provide information that differs from visual appearance. LiDAR point-cloud distributions have been used for orchard inter-row localization [62], while multilevel point-cloud features and attention support crown–trunk separation in nursery trees [63]. Vehicle vibration acceleration has also been analyzed using principal component analysis, genetic-algorithm optimization, and an extreme learning machine to classify ground types [64]. These examples demonstrate alternative sensing pathways. Their complementary information creates opportunities for integration, although separately successful sensing methods do not establish the performance of a fused system. Table 3 summarizes representative perception tasks, validation contexts, and evaluation requirements.

Combining these inputs requires attention to the different causes of measurement variation. Vibration can change with terrain, speed, and payload, while geometric measurements depend on sensor placement and calibration. Platform motion, including payload sloshing in spraying UAVs, further complicates sensing and control [65]. Multi-sensor integration and closer coupling between perception and navigation have consequently emerged as related research directions [66,67]. Integration should preserve the spatial and temporal relationships among measurements and account for changes in their reliability. Otherwise, additional inputs may introduce inconsistencies into the estimated scene or machine state.

Across the navigation studies, the relevant question is whether richer information produces more reliable navigation under the conditions encountered by the machine. Evaluation should connect perception errors and latency with path deviation, intervention frequency, and task completion. For fused systems, matched comparisons with individual inputs and tests involving degraded or missing measurements can establish the contribution of sensor complementarity. These considerations also inform condition-monitoring systems, where distinguishing changes in the environment from changes in the machine requires reliable interpretation of sensor information. Direct evidence for machinery fault diagnosis is examined in Section 7.

## 6. Sensor-Informed Automation and Operational Monitoring

### 6.1. Autonomous Navigation System Architecture

Autonomous navigation requires sensor information to be converted into estimates of machine position, surrounding geometry, and task-relevant boundaries [68]. A common architecture combines Real-Time Kinematic Global Navigation Satellite System/Inertial Navigation System localization with visual crop-row detection and LiDAR point-cloud processing [69]. These inputs provide position and motion estimates, visual task cues, and geometric information, respectively. Their usefulness depends on the operating environment and the consistency of the information supplied to planning and control. Sensor configuration and integration therefore need to account for differences among open fields, orchards, and greenhouses. Figure 6 summarizes the main modules and information flow.

In greenhouses, orchards, and warehouses with unavailable or unreliable global navigation satellite system (GNSS) signals, simultaneous localization and mapping (SLAM) supports autonomous navigation. For warehouse-to-road transitions, improved Lightweight and Ground-Optimized LiDAR odometry and mapping, combined with normal distribution transform-inertial measurement unit-unscented Kalman filter fusion, reduced mapping errors [70]. For orchard inspection, LiDAR and inertial measurement unit (IMU) sensing were integrated with improved A* and dynamic window approach (DWA) planning. The robot achieved a docking lateral deviation of approximately 0.16 m and avoided static obstacles [71]. To correct accumulated LiDAR-inertial odometry drift, AprilTag visual markers provided absolute pose observations in place of GNSS. At 3 m spacing, mean localization error and mapping root mean square error reached 0.041 m and 0.057 m, respectively [72]. These studies demonstrate the feasibility of local navigation under GNSS-denied conditions. However, marker deployment costs and insufficient validation under extreme illumination or severe occlusion remain barriers to wider application.

Task-specific navigation extends beyond positioning and obstacle avoidance to integrate operational perception and human–machine interaction. For combine harvesters, tightly coupled binocular SLAM and IMU localization supports crop-boundary guidance extracted through homomorphic filtering and edge detection. Single-neuron proportional-integral-derivative (PID) steering control maintains low lateral deviation and high cutting-width efficiency across operating speeds [73]. For complex or high-risk scenarios, UAV mapping and virtual reality closed-loop control integrate local feature transformer matching into structure-from-motion reconstruction. This improves reconstruction in low-texture regions and provides detailed 3D environments to support operators’ spatial understanding [74]. Together, these approaches extend navigation toward task-specific autonomous control and immersive human–machine interaction.

### 6.2. Route Planning

Path planning in dense orchards and greenhouses must ensure efficient and safe operation within confined spaces. Dense and changing obstacles require rapid planning and precise obstacle avoidance [75]. To address canopy-induced localization drift, tightly coupled SLAM suppresses long-distance axial drift, while Bayesian generalized kernel inference assesses traversability from 3D point clouds at sub-meter resolution. The resulting risk-weighted 2D occupancy grids enable rapidly exploring random tree planning to generate feasible paths within 0.6 s [76]. For trajectory optimization, Voronoi-derived centerlines guide D* Lite planning, while hybrid A* smooths curvature subject to kinematic constraints, keeping mean path deviation within 0.06 m [77].

Beyond local obstacle avoidance, planning must address field coverage and coordination between mobile bases and manipulators. Combining UAV-derived global information with historical operating logs, Markov-chain state-transition models support adaptive machinery commands, achieving an operational distance utilization rate of 72.56% [78]. For greenhouse harvesting, deep deterministic policy gradient supports coordinated base and arm motion within dynamic projection maps. This approach reduced invalid stops by 75% for dual-arm harvesting robots operating among dense targets [79].

These studies translate estimated position, traversability, and task information into feasible motion plans. Planning performance consequently depends on the spatial accuracy and update frequency of its inputs, as well as the representation of machine constraints. Evaluation should examine whether routes remain executable when localization drifts, scene information becomes outdated, or operating conditions change. This connects perception quality with planning outcomes without attributing all improvements to the planning algorithm alone.

### 6.3. Path Tracking and Motion Control

Path tracking precision is a key indicator of autonomous AM operating quality. Variations in soil moisture and softness cause wheel slip and sideslip, leading to deviations from planned trajectories. To improve on geometric error feedback, recent approaches incorporate slip parameters into kinematic models and use online observers to estimate and compensate for ground disturbances [80].

The control problem concerns how measured or estimated states are used to regulate motion under disturbances. State observers, parameter adaptation, and sensor fusion can contribute at different points in this process but should not be treated as interchangeable methods. Evaluation should connect tracking error with feedback quality, actuator effort, and task performance under the stated operating conditions.

The following subsections examine three complementary aspects of motion control. Section 6.3.1 addresses robust disturbance rejection and chattering suppression through sliding mode control (SMC). Section 6.3.2 examines online parameter adaptation and predictive constraint handling, while Section 6.3.3 addresses dynamic coupling and coordinated control between the chassis and functional implements.

#### 6.3.1. Robust Disturbance-Rejection and Chattering Suppression

Achieving high-precision path tracking requires a balanced trade-off between fast error convergence and control signal smoothness. To address non-linear perturbations induced by unstructured farmland, the establishment of sophisticated kinematic models paired with high-order observers serves as the foundation for enhancing systemic robustness. For instance, within the kinematic architecture of four-wheel independent steering robots, the explicit integration of lateral/longitudinal slip velocities and slip angles—coupled with a second-order extended disturbance observer that requires no prior knowledge of disturbance upper bounds and an improved sliding surface—can effectively mitigate initial overcompensation and significantly reduce the mean path deviation under paddy field conditions [81]. However, the substantial computational overhead associated with high-order observers may present challenges in meeting the frequency requirements for real-time control on embedded platforms with limited processing power. This remains a primary constraint for the engineering deployment of such methodologies.

To meet the strict deviation tolerances of AM, recent sliding-mode control studies have progressively combined prescribed-performance constraints, disturbance observation, and higher-order continuous control. Prescribed-performance control (PPC) with an exponential reaching law transforms the tracking error so that both transient and steady-state responses remain within predefined bounds, while a dual-loop architecture combining inner-loop sliding-mode control (SMC), a nonlinear disturbance observer, and outer-loop model predictive control (MPC) improves the convergence of position and heading errors in tractor-trailer systems [82,83]. However, rigid error constraints may cause control-input saturation and transient instability under severe disturbances.

To improve actuator compatibility and reduce the chattering associated with conventional SMC, higher-order sliding-mode and super-twisting strategies have been developed. Second-order SMC generates continuous control inputs to attenuate high-frequency switching effects [84]. Robust exact differentiation improves state reconstruction when measurements are unreliable [85], while fixed-time generalized super-twisting control addresses slip-induced underactuation and guarantees convergence within a predefined time interval [86]. Fast super-twisting control combined with time-varying bandwidth observers further suppresses observer peaking and provides an explicit convergence-time bound [87]. Collectively, these studies show a progression from constraining tracking errors to smoothing control inputs and guaranteeing finite-time convergence. Nevertheless, the performance of these methods remains sensitive to controller gains and disturbance characteristics. When the machine moves across substantially different terrains, repeated parameter retuning may be required, which limits cross-scenario transferability and motivates the adaptive control strategies discussed next.

Building on these limitations, adaptive gain mechanisms have been introduced to match control intensity to unknown disturbance levels without requiring prior knowledge of disturbance bounds. A positive-semidefinite barrier function can prevent excessive gain growth caused by disturbance overestimation and keep the sliding-mode variables within a predefined region [88]. For dynamically coupled models, combining preview lateral deviation with adaptive second-order SMC improves uncertainty handling in unstructured terrain and reduces the root mean square error across different operating scenarios [89].

A related line of research uses learning-based compensators to estimate mechanical interaction residuals online. Recursive least-squares extreme learning machines have been used to perceive external disturbances in tracked harvesters [90], while radial basis function neural networks with power-rate reaching laws compensate for sideslip interference in paddy fields and reduce lateral deviation to the centimeter level [91]. Similar learning-based compensation has also been applied to the visual servoing of harvesting robots, where fuzzy neural networks approximate the equivalent control law without requiring an accurate dynamic model [92]. These methods combine disturbance estimation with corrective control through different model-based or learning-based mechanisms. Their practical performance depends on whether the estimated disturbances remain informative when terrain, payload, and actuator behavior change. Controller tuning, estimation error, and computational requirements should therefore be assessed together. The relevant outcome is stable motion regulation under specified conditions, with the contribution of each estimation or compensation component made explicit.

#### 6.3.2. Online Parameter Adaptation and Predictive Constraint-Handling

Traditional geometric steering laws, such as Stanley and pure pursuit, generally use fixed gains and preview parameters, which can increase tracking errors during high-curvature maneuvers and under changing soil-slip conditions. Recent adaptive extensions have introduced sideslip estimation, curvature compensation, fuzzy gain optimization, dynamic look-ahead selection, and angular-velocity feedback to improve tracking performance across different terrains and operating tasks [93,94,95,96,97]. However, these methods remain largely dependent on kinematic parameter tuning and may provide limited stability guarantees under abrupt terrain transitions or actuator saturation. Parameter adaptation can compensate for deviations observed during calibration, but it does not necessarily identify their physical causes. Similar tracking errors may result from different combinations of wheel slip, terrain curvature, payload variation, and actuator delay, while requiring different corrective actions. Adaptive geometric controllers may therefore remain vulnerable when multiple disturbances occur simultaneously or when environmental changes exceed the response speed of the update mechanism.

Nonlinear adaptive controllers further address disturbance rejection, parameter evolution, and anti-saturation requirements. Strong-tracking Kalman filtering combined with adaptive anti-windup PID control has been used to improve convergence under sensor noise and intense interference [98]. Back-propagation neural networks and soft-switching sliding-mode observers have been applied to optimize backstepping parameters and compensate for time-varying slip disturbances [99]. For tractor-trailer systems, fuzzy adaptive backstepping controllers formulated in the Frenet frame have reduced trajectory deviations during U-turn maneuvers, while adaptive heading-feedback and nonlinear PID control have improved header utilization in autonomous paddy-field harvesting [100,101]. Nevertheless, increasing the number of adaptive parameters complicates controller tuning, and delayed parameter updates may degrade transient performance under rapidly changing unmodeled dynamics.

MPC provides a complementary predictive framework by optimizing control sequences over a finite horizon while explicitly handling constraints and predicted disturbances. For four-wheel independent-drive agricultural vehicles, a high-order disturbance-observer-enhanced explicit nonlinear MPC has enabled real-time estimation and compensation of slip velocity and slip-angle disturbances [102]. However, the practical advantage of MPC depends on model fidelity, prediction-horizon selection, constraint formulation, solver convergence, and onboard computational resources. Its performance may deteriorate when soil or vehicle parameters fall outside the prediction model, particularly on embedded platforms with limited processing capacity. Comparisons across studies are also hindered by inconsistent reporting of tracking accuracy, computation time, actuator constraints, and disturbance conditions. The key challenge is therefore to maintain sufficiently fast, stable, and computationally bounded adaptation during abrupt transitions between heterogeneous field conditions.

#### 6.3.3. System-Level Dynamic Coupling and Multi-Component Cooperative Control

Whole-machine control requires coordination between chassis motion and functional components, including headers, threshing drums, and spraying units. Changes in harvester feed rate, for example, alter tractive resistance and affect trajectory tracking. Such coupling links motion control directly to implement performance and overall work quality.

Cooperative control frameworks integrate reinforcement learning with agronomic constraints and distributed multi-agent architectures to support trajectory optimization and robust perception [103]. For distributed-drive electric tractors, hierarchical coordination-consensus control combines fixed-time extended state observers with non-singular terminal SMC to synchronize wheel speeds, mitigating slip and soil compaction [104]. For tractor-trailer units and harvesters operating in confined spaces, leader-follower control combines SMC, a fast power-rate reaching law, and radial basis function neural networks. This approach compensates for uncertainties associated with payload variation and soil conditions [105]. However, delayed or inaccurate information can propagate across vehicles and components, allowing local tracking corrections to compromise implement stability or synchronization. Evaluation should therefore include packet loss, asynchronous sensing, actuator faults, and abrupt load changes, with explicit assessment of degraded-mode operation and fault containment.

Within grain harvesters, dynamic modeling supports coordinated control of coupled threshing and cleaning processes. PSO combined with a wavelet neural network enables state-space identification of a three-input, three-output system for multivariable control [106]. YOLOv4-based visual feedback integrated with MPC regulates entrainment loss and impurity content, reducing system response latency to less than 3 s [107]. For electrically driven threshing drums, recursive least squares with a forgetting factor identifies inertia online to update proportional-integral control parameters. This approach reduced load-induced speed deviations by more than 55% [108]. However, accurate model fitting does not guarantee stable control under unseen loads. Improvements in drum-speed stability should therefore be assessed alongside grain loss, impurity content, fuel consumption, and structural loading. Models should be evaluated by their ability to support stable multi-objective control across operating conditions.

Beyond internal harvesting processes, coordinated control must also adapt implement operation and travel speed to changing terrain and task requirements. For header-height measurement, Kalman filter fusion combined with fuzzy decision-making adjusted filter covariance and improved detection stability on uneven ground [109]. Grey prediction PID control used feedback from the profiling mechanism to anticipate terrain undulations, constraining the mean header deviation to within 21 mm [110]. For furrows and lateral slopes, multi-sensor MPC combines a third-order state-space model with global navigation satellite system real-time kinematic (GNSS-RTK) and inertial navigation data to correct machine attitude. This approach reduced the coefficient of variation in stubble height relative to conventional algorithms [111]. For tiger nut harvesting, backstepping-PID control adaptively coordinates excavation depth and rotational speed [112]. At the whole-machine level, LSTM-based operating-speed prediction combined with incremental PID hydraulic control improved operational efficiency by 9.1% while maintaining required work quality [113].

Across these applications, feedback describes different aspects of operation, including wheel motion, grain-flow quality, drum speed, header position, and machine attitude. Coordinated control depends on relating these variables to the components and processes being regulated. Evaluation should distinguish improvements arising from measurement fusion, online model updating, and controller design. Reporting the timing and operating conditions of the feedback signals would also clarify whether local improvements persist during coupled whole-machine operation.

### 6.4. Intelligent Harvesting

Intelligent harvesting uses information about both crop attributes and machine operating conditions to guide actuation. In densely occluded canopies, dual-threshold segmentation of three-dimensional point clouds combined with adaptive clustering has been used to resolve overlapping targets [114]. This provides geometric information relevant to the limitations of conventional visual monitoring [115].

Internal process monitoring addresses a different set of variables. Multimodal data fusion has been applied to impurity estimation in grain flow [116]. WOA-BP-based loss monitoring [117] and differential compensation for vibration interference in grain-flow sensing [118] address measurement performance through different approaches. These examples should be distinguished according to whether they combine complementary inputs, estimate an operational variable, or suppress interference affecting a measurement.

A more direct application to abnormal-state identification combines strain, vibration, and rotational-speed signals in a genetic algorithm–SVM model for blockage detection in a potato–soil separation device [119]. These signals describe loading, dynamic response, and rotational behavior, providing complementary observations of the operating process. Establishing the benefit of their combination requires comparisons with individual inputs under matched conditions. This example connects harvesting-process monitoring with the fault-diagnosis discussion in Section 7. Figure 7 summarizes the broader pathway from perception to adaptive harvesting actuation.

At the end-effector level, the central challenge resides in converting perception outputs into precise mechanical actions in real time. Although contemporary fruit and vegetable harvesting robots have achieved notable advances in recognition accuracy, the low operational efficiency of single-arm configurations remains a critical bottleneck [120]. Reinforcement learning algorithms grounded in Markov game theory have been introduced into multi-arm systems, reducing operational cycle times through dynamic task allocation and collision-avoidance planning [121]. Dual-arm cooperative kinematic modeling provides a theoretical foundation for handling complex manipulation tasks involving unstructured agricultural objects [122]. Beyond efficiency enhancement, the physical interaction between end-effectors and delicate crops necessitates a balance between grasping success rate and minimal damage. By constructing a Burgers model that captures the viscoelastic characteristics of asparagus and coupling it with neural network prediction, real-time optimal regulation of gripping force has been achieved [123]. For mulberry branch cutting insertion with heterogeneous germination states, a delta robot system integrating multi-view machine vision with a lightweight tiny-YOLO model has demonstrated high-speed, precision operation capability in aeroponic cultivation environments [124]. Furthermore, algorithmic innovation in operational modes plays an equally pivotal role; for instance, a combined multipoint picking scheme proposed for tea harvesting replaces conventional single-point plucking by solving an optimal coverage set problem, thereby substantially eliminating redundant motion at the geometric path level [125]. Nevertheless, the contradiction between the high computational overhead imposed by complex cooperative algorithms and the limited processing capacity of agricultural embedded platforms confines most current optimization strategies to simulation or short-duration trials, with validation under all-weather, high-intensity field operations still lacking. This underscores that algorithmic refinement alone is insufficient to resolve all operational challenges, and a holistic approach encompassing mechanical structural design and agronomic compatibility must be concurrently pursued.

Perception and control algorithms need to be implemented on complete machine platforms and adapted to specific crops and terrain conditions. In large-scale cereal production, combine harvester upgrading depends on coupling multi-sensor fusion navigation with whole-machine attitude control to mitigate operational deviations from narrow rows and terrain undulation in soybean–maize strip intercropping [126]. A broader review of intelligent grain-harvester systems discusses their applications to operational quality and energy management [127]. Specialized equipment development shows differentiated adaptation to specific landforms and crops. For aquatic environments, a catamaran-based system with an Improved RRT algorithm has addressed water disturbance and path planning in Brasenia schreberi harvesting [128]. In hilly and mountainous regions, a crawler-type potato harvester incorporating residual film recovery and multi-stage soil-tuber separation devices has overcome the trade-off between operational resistance and tuber damage in cohesive soils through kinematic parameter matching of key components [129]. A self-propelled baby bok choy harvester based on a programmable logic controller and electric chassis achieves clean-energy operation and precision cutting within confined spaces through closed-loop control [130].

Overall, the majority of existing integrated solutions exhibit a high degree of customization, lacking standardized modular platform architectures transferable across diverse crops and geographical regions. Future efforts should prioritize standardized, modular interfaces among sensing, control, and mechanical subsystems, including consistent timestamps and signal-quality information to support the reuse of measurements for operational control and condition monitoring across harvesting platforms.

Thus, intelligent harvesting is distinguished from Precision in-field management by its product-removal objective: harvesting systems organize perception, control, and actuation around crop detachment, loss reduction, and throughput, whereas in-field management applies similar pipelines to crop-state intervention and resource-input regulation, including spraying, weeding, and transplanting.

### 6.5. Precision In-Field Management

The intelligent core of in-field management lies in adjusting operational parameters such as pesticide and fertilizer application according to the actual growth status of crops, which requires establishing a complete pipeline from crop state perception to adaptive regulation of operational parameters. To address the challenge of low spray utilization in orchards, a multivariable robot integrated with 3D LiDAR extracts canopy volume using an improved Density-Based Spatial Clustering of Applications with Noise algorithm and synchronously regulates flow rate, air volume, and droplet size, achieving a substantial reduction in chemical drift compared with conventional spraying modes [131]. For potted plants, a lightweight shufflenet-YOLOx nano-efficient channel attention model combined with leaf phenotypic information enables differentiated fertilization decisions based on the leaf area coefficient, validating the high adaptability of double-sided targeted spraying [132].

The usefulness of these perception outputs depends on their reliability under changing illumination, canopy occlusion, and machine motion. Similar constraints arise in precision weeding, where target position must be translated into a timely intervention while avoiding nearby crops.

Precision weeding systems integrate CNN/Transformer-based real-time detection models with multi-source sensing data to actuate terminals, cutting herbicide use by 30-90% in trials [133]. To address the slow actuator response and low control accuracy in intra-row weeding, a scheme combining YOLOv5s-transformer with Kalman filtering was adopted [134]. However, as operational speed increases, both weeding precision and crop damage rates deteriorate, indicating that existing control logic remains limited in compensating for dynamic errors induced by high-speed movement. Reconciling enhanced operational efficiency with actuator reliability constitutes the central challenge in transitioning such systems from experimental prototypes to practical field deployment.

These speed-dependent errors highlight the importance of maintaining correspondence between a sensed target and the subsequent actuator response. During machine motion, acquisition, processing, and actuation delays can make an initially accurate target estimate outdated by the time an intervention occurs. Terrain-induced vibration further complicates this relationship. Reliable operation therefore requires coordinated perception and execution, with target information interpreted in relation to machine motion and response timing.

The information required for this coordination also depends on the operation and crop arrangement. In elevated strawberry transplanting, PLC-controlled manipulation sequences coordinate seedling extraction and planting operations to meet specific orientation requirements [135]. This example illustrates how the required action depends on the configuration of the object being handled. In soybean–maize strip intercropping, overlapping canopies obscure target plants and complicate selective operations [136]. Here, the limitation concerns whether the target can be adequately observed before an action is selected. Together, these examples distinguish two requirements: obtaining sufficient target information and maintaining its correspondence with the intended action.

Multi-source sensing can support these requirements when complementary observations address missing target information or relate that information to the machine’s operating state. Fusion should therefore be designed around a specific information gap, with the contribution of each input and its spatial and temporal alignment made explicit. Additional inputs may offer limited benefit when they share the same occlusion or introduce inconsistent observations. Evaluation should compare fused and individual inputs under matched occlusion and operating-speed conditions, linking estimation errors and response delays to application accuracy, crop damage, and task completion. Such comparisons would clarify when sensor fusion improves field-operation reliability and when degraded information requires reacquisition or conservative operation.

## 7. Sensor-Based Intelligent Operation and Maintenance

AM operates continuously under harsh field conditions characterized by intense vibration, high dust concentrations, and variable loads. Unexpected failures of critical components not only disrupt operations but may also lead to serious safety incidents. Conventional scheduled maintenance approaches frequently encounter either excessive servicing waste or the risk of under-maintenance, and struggle to accommodate the demands of high-intensity cross-regional operations. AI technology offers new pathways for condition monitoring and fault diagnosis in AM [137,138,139]. Within this context, a central consideration is how complementary sensor measurements can support the identification of abnormal machine states under noisy and variable operating conditions. Intelligent O&M therefore requires reliable signal acquisition, extraction of fault-related information, and the translation of diagnostic results into operational or maintenance decisions. Key challenges include distinguishing incipient faults from load-induced signal variations, assessing component health where sensor coverage is limited, and maintaining diagnostic reliability when sensing quality deteriorates. Machine-learning-based predictive maintenance models have also been explored to assess the health and deterioration of robotic components [140]. The general architecture of an AI-driven fault-diagnosis and prognostics system is illustrated in Figure 8.

Fault feature extraction and noise suppression constitute the primary step in intelligent O&M. During harvester operation, high-frequency vibrations from the threshing drum and load fluctuations at the header often mask incipient signals associated with bearing wear or blockage onset. To extract meaningful features from intense background noise, researchers typically employ empirical mode decomposition or variational mode decomposition to first decompose mixed signals into intrinsic mode functions for environmental noise filtering, followed by CNNs or recurrent neural networks to capture the spatiotemporal evolution patterns of fault features. This approach has demonstrated notable effectiveness in diagnosing mechanical damage such as motor bearing faults, achieving experimental accuracies of up to 98% [141]. The scarcity of field fault samples remains a persistent bottleneck constraining model generalization capability. Beyond utilizing generative adversarial networks for dataset augmentation, optimizing SVM parameters through improved particle swarm optimization (IPSO) has proven effective in enhancing classification performance under small-sample conditions [142]. However, the efficacy of existing algorithms is highly contingent upon well-structured data collected in laboratory settings. Under actual field operating conditions, nonlinear signal drift induced by variations in crop moisture, density, and random impact loads frequently causes substantial degradation in model performance. For fusion-based diagnosis, comparisons with individual-sensor inputs under matched operating conditions are needed to establish the contribution of complementary measurements. Tests under changing loads and added noise should further distinguish improvements in classification accuracy from robustness to degraded sensing.

**Figure 8:**
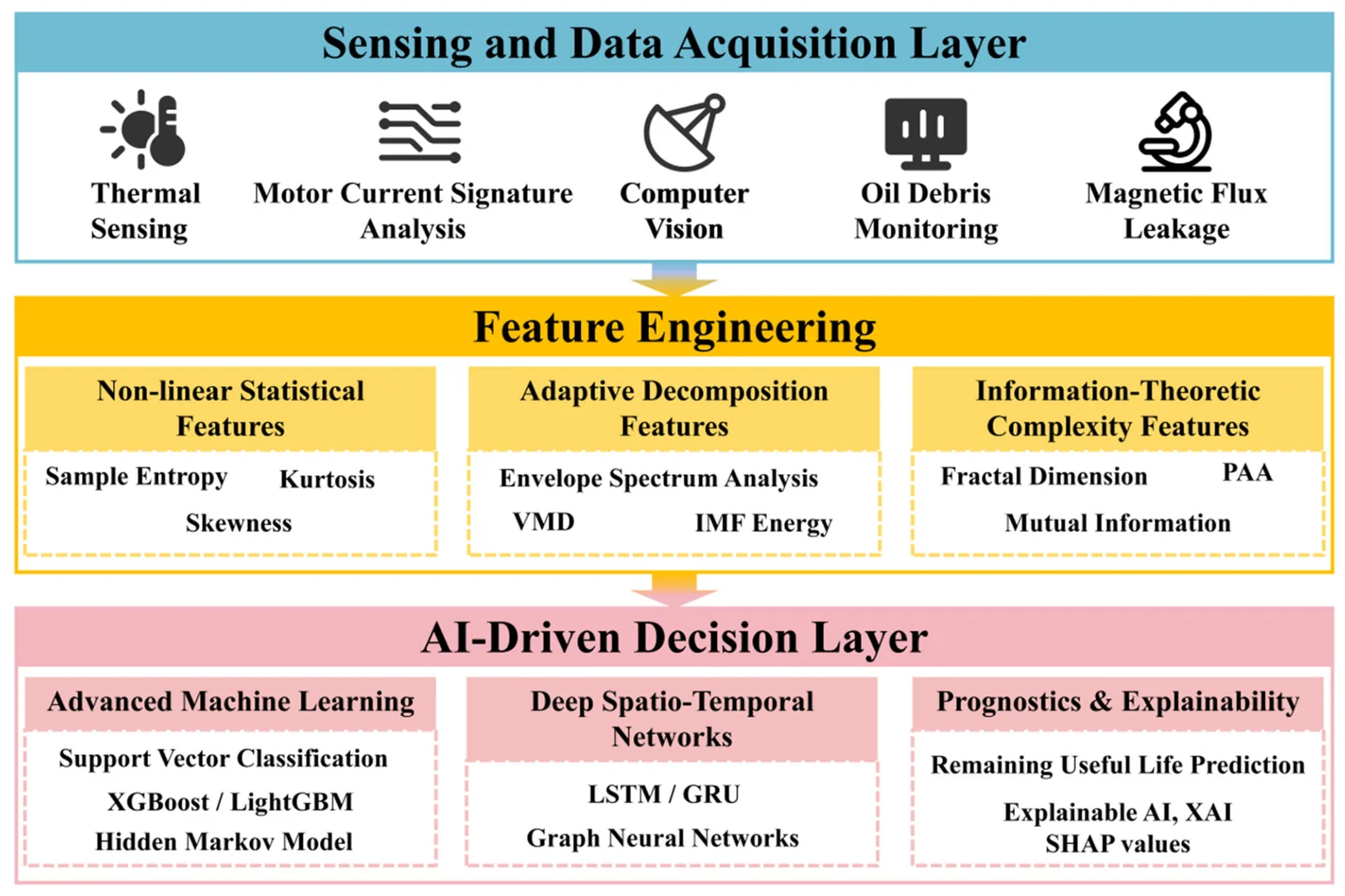
Architecture of the AI-driven fault diagnosis and prognostics system [142].

Structural life prediction and digital twin-driven repair offer novel approaches to addressing the challenges of sensor installation at critical locations and rapid post-damage recovery. Harvester frames and welded joints are subjected to prolonged irregular impact loading, and conventional fatigue analysis, which relies primarily on linear cumulative damage theory, struggles to accurately capture real-time loads at structurally critical regions. Given the harsh harvesting environment and spatial constraints, physical sensors often cannot be deployed in high-temperature, intensely vibrating core zones, or their service life is significantly shortened by severe vibrations. Dynamic load spectra generated through finite element analysis and multi-body dynamics simulations enable the estimation of stress responses at inaccessible locations, thereby providing data support for life assessment [143]. Building upon this foundation, digital twin frameworks integrate intelligent diagnostics with additive manufacturing technologies. Through real-time computation of damage propagation trends using high-fidelity models, such frameworks not only facilitate maintenance planning but also enable rapid repair of damaged components via techniques [144]. Although this framework achieves full life-cycle coverage, the online solving speed of high-fidelity multi-physics models remains substantially below the requirements for real-time decision-making, representing the principal bottleneck in advancing digital twins from offline analysis to online control.

Multi-sensor monitoring provides a link between fault detection and operational intervention. Rotational-speed measurements from Hall-effect sensors installed on multiple harvesting components can be converted into slip-rate indicators and jointly processed by an IPSO-SVM model to assess clogging risk. Monitoring several transmission components provides complementary information that may be missed by single-component load feedback. Combining the resulting risk estimates with feeding-rate information enables fuzzy control of travel speed, linking multi-component sensing to corrective action [145]. In energy management for hybrid harvesters, employing neural networks to approximate the globally optimal strategy of dynamic programming reduces the single-step computation time for engine power allocation to 2 ms, achieving approximately 11% energy savings while maintaining real-time responsiveness [146]. By shifting computationally intensive training processes to the offline stage and executing only lightweight inference online, embedded controllers are enabled to meet millisecond-level response requirements. However, such methods inherently depend on the distributional coverage of training data. When sensors experience drift or encounter extreme operating conditions absent from the training set, strategy networks lacking explicit physical constraints may generate unreasonable control commands. Therefore, the fault tolerance performance of these systems under unexpected operating conditions still requires validation through long-term field trials. These examples show that intelligent O&M should extend beyond isolated fault classification or control optimization. A complete maintenance-oriented system must also address data scarcity, component degradation, hydraulic-system controllability, physical-state evaluation, and the conversion of health information into timely maintenance decisions.

### From Fault Diagnosis to Condition-Oriented Maintenance

The existing literature indicates that intelligent O&M are gradually moving from isolated fault classification toward condition-oriented assessment. However, this transition remains incomplete. Current studies provide methodological foundations for estimating component health, detecting degradation, and linking machine condition to operational control, but they rarely establish a complete maintenance decision process.

Data scarcity is one of the main constraints on intelligent maintenance in AM. One-shot transfer learning has been proposed for bearing-component fault diagnosis under conditions in which only a very small number of target-domain samples are available [147]. This direction is relevant to AM because complete failure datasets are difficult to obtain and the same component may operate under different speeds, loads, temperatures, and seasonal conditions. Nevertheless, the reliability of transferred diagnostic features should be tested under changes in machine configuration, sensor position, operating load, and environmental conditions.

Fault diagnosis should also distinguish between an isolated abnormal event and progressive component degradation. A hybrid VGG-LSTM model has been developed for hydraulic-pump wear-defect detection by combining feature extraction with temporal-sequence modeling [148]. Such a model can provide a basis for health assessment and early warning because it considers both the characteristics of the measured signal and the temporal evolution of wear. In AM, however, the practical value of this approach depends on whether the model remains reliable under changing hydraulic pressure, load demand, temperature, sensor degradation, and intermittent operation.

Hydraulic actuation provides an important connection between machine O&M. Independent metering control systems have been reviewed as a means of improving flow regulation, pressure control, energy efficiency, and load adaptation in mobile machinery [149]. These control variables are also relevant to maintenance because abnormal flow response, pressure fluctuation, actuator synchronization, or excessive control effort may indicate valve degradation, leakage, pump wear, or actuator impairment. Therefore, future intelligent maintenance systems should combine diagnostic signals with operational control information rather than treating component health and machine operation as separate problems.

Physical information and closed-loop evaluation provide another potential route for improving maintenance reliability. A closed-loop identification framework for rubber mixing incorporated process-related physical information into a deep-learning model and linked state perception, process evaluation, and feedback adjustment [150]. Although the application was not AM, the methodological implication is relevant: health indicators should be evaluated against physically meaningful machine states and operational consequences instead of statistical prediction accuracy alone. Similar principles could be applied to AM by constraining health assessment with load, pressure, speed, vibration, temperature, and task-quality information.

Taken together, these studies provide the foundation for data-efficient diagnosis, degradation monitoring, hydraulic-system assessment, and physically constrained health evaluation. They do not yet constitute a complete maintenance-management framework. The remaining challenge is to convert these health estimates into remaining-useful-life prediction, maintenance timing, repair planning, and lifecycle decisions.

## 8. Challenges and Future Perspectives

The foregoing literature shows that AI has been applied across perception, control, field operations, and maintenance in AM [151,152,153]. Most results have yet to move beyond controlled, single-technology validation. The main barriers to field deployment are therefore not limited to whether individual algorithms work in principle. They arise from unresolved mismatches between algorithmic complexity and onboard hardware, laboratory training conditions and real farming environments, isolated module development and whole-machine operation, and performance optimization and safe, predictable behavior. To ground these trade-offs, Table 4 summarizes the core capabilities, inherent bottlenecks, and best-suited scenarios of the primary AI paradigms discussed. Four closely related challenges currently constrain the transition from experimental validation to dependable field deployment: computational limitations, poor cross-scenario generalization, fragmented system integration, and insufficient safety assurance.

### 8.1. Computational Feasibility, Model Generalization, and Cross-Scenario Adaptation

Regarding computational constraints and real-time deployment, multimodal fusion perception, MPC, and digital-twin-based online simulation all impose substantial computational demands. The embedded platforms available for field use, by contrast, operate under tight ceilings on processing throughput, power draw, and heat dissipation. Cloud-edge-device architectures offer one strategy for managing this tension by distributing computational tasks according to their latency requirements. Studies in farmland monitoring and autonomous navigation have shown that such architectures can reduce latency while preserving diagnostic performance [154,155]. However, the critical design question is not how much computation can be offloaded, but which functions can be offloaded without making operational safety dependent on network availability. Safety-critical functions should remain locally executable, whereas model retraining and fleet-level optimization can tolerate delayed cloud processing. Future studies should report worst-case latency, energy consumption, thermal throttling, and performance after communication loss, rather than average inference speed alone. Yet tiered architectures alone do not settle the matter. Terrain features and shifting working distances destabilize wireless links, and prolonged vibration, airborne dust, and swings in temperature and humidity steadily erode the dependability of edge hardware itself. Progress therefore needs to occur on two fronts. At the algorithmic level, compact models, event-driven inference, and the removal of redundant network operations can reduce unnecessary computation. At the hardware level, edge devices must be ruggedized against vibration, dust, and environmental fluctuations. If either front is neglected, even a well-performing algorithm risks becoming inoperative in the field because of hardware degradation or a dropped communication link.

Beyond computational feasibility, reliable field deployment depends on whether models maintain their performance when agricultural conditions change. Model performance often deteriorates when systems are transferred beyond the conditions used for development. Agricultural environments are shaped simultaneously by crop physiology, weather, soil characteristics, and management practices, which are difficult to reproduce comprehensively under laboratory conditions. As a result, many existing training datasets represent only a limited subset of the variability encountered in agricultural environments. Closing this gap calls for action on two levels. On the data side, the community needs openly accessible, multi-region, multi-crop benchmark datasets built around shared annotation conventions and evaluation protocols, so that results from different groups can be meaningfully compared. On the methodological side, encoding domain knowledge—soil mechanical behavior, crop phenological trajectories—as hard or soft constraints during training can anchor model outputs within physically plausible bounds when the system encounters conditions absent from its training set, improving robustness without demanding ever-larger labeled corpora. Visual-language pretrained models combined with dynamic calibration offer another route to unsupervised domain adaptation [156]. Yet online adaptation introduces a second problem: a model may improve its local accuracy while becoming harder to reproduce and verify. Evaluation should extend beyond adaptation accuracy on a familiar target domain to leave-one-region-out, leave-one-season-out, and leave-one-crop-out testing combined with uncertainty calibration. A related but often overlooked issue is whether control gains, observer tuning, and path-planning thresholds calibrated for one machine-field combination can transfer reliably to another; work on this front remains sparse. Thus, stable perceptual accuracy does not necessarily imply stable control performance, because identical outputs may produce different consequences on machines with different geometries, actuator dynamics, or payloads.

### 8.2. System Integration and Safety Assurance

Turning to system integration and collaborative operation, the preceding review shows that chassis design, end-effector engineering, perception algorithms, and control strategies have each advanced considerably, but largely within separate research silos. Unified classification-design-optimization-application frameworks seek to integrate end-effector design with motion control and trajectory optimization, providing important theoretical groundwork [157]. However, actual machines still lack adopted specifications that link the outputs of perception modules to the inputs required by controllers. Subsystems are often connected through case-by-case engineering compromises, while inter-module parameters, timing, and uncertainty remain insufficiently coordinated.

This fragmentation occurs both within individual machines and across fleets. For example, header-height adjustment and forward-speed control may operate without real-time coordination. At the multi-machine level, data formats, task-allocation logic, and communication protocols may differ across platforms. Proposals have extended coordinated operation from individual tasks to the full tillage-planting-management-harvest chain [158]. This vision requires conventions for data exchange, control handshakes, and shared metrics. Controllers need timing, coordinate frame, uncertainty, and degradation status for each perception estimate. The key challenge is establishing explicit timing and uncertainty contracts between modules, validated by end-to-end error-propagation tests. Without them, each integration starts from scratch.

These integration challenges are directly connected to safety assurance and model trustworthiness. As data-driven methods become more widely used for perception, control, and predictive maintenance, their behavior outside the statistical domain represented by the training data remains poorly characterized. The system may then lack a reliable mechanism for detecting or mitigating such outputs. Physical constraints can be incorporated into network architectures to limit outputs to feasible ranges. Tiered fallback strategies, ranging from conservative operation to safe shutdown, can be activated when perception or control integrity is compromised. Explainable artificial intelligence methods may improve the transparency of deep-learning decisions [159]. However, explanation should not be treated as verification. Identifying the inputs that influence a prediction does not establish that the prediction is causally valid or operationally safe.

Digital twins provide an additional testing environment in which boundary-case scenarios can be rehearsed before they are encountered in the field. Their adoption in AM remains constrained, however, by the cost of constructing high-fidelity virtual models and maintaining synchronization with their physical counterparts in real time [160]. Their evidential value therefore depends on both model fidelity and scenario coverage. A digital twin that omits relevant failure modes may create false confidence rather than reduce risk. Safety evaluations should consequently report false-negative rates for out-of-distribution detection, the time required to reach a safe state, and residual performance under partial sensor or communication failure.

Computational constraints limit the deployment of sophisticated models on field machines, while narrow or unrepresentative datasets reduce their robustness under changing conditions. In parallel, the absence of shared interfaces prevents individually validated modules from operating reliably as an integrated system. These limitations become most consequential when information and uncertainty propagate through the complete machinery chain, from sensing and perception to control, actuation, task execution, and maintenance. System reliability should therefore be evaluated across computational feasibility, cross-scenario robustness, interface compatibility, and safety assurance rather than within isolated modules.

### 8.3. End-to-End Information and Error Propagation Across the Machinery Chain

The reviewed studies indicate that the performance of intelligent AM cannot be evaluated adequately by examining perception, control, actuation, and maintenance as independent modules. A complete machine-level chain consists of sensing, perception, decision-making, control, actuation, task execution, condition monitoring, and maintenance decision-making. Each stage transforms the information produced by the previous stage, while also introducing uncertainty that may propagate downstream.

To make these interactions explicit, Table 5 summarizes the information flow from sensing to maintenance decision-making. For each stage, the table identifies the typical inputs, the resulting AI output, the main sources of error, the downstream consequence of error propagation, and the engineering indicators that should be reported. This framework emphasizes that an AI result should be evaluated not only by its local prediction accuracy, but also by how uncertainty at one stage affects control behavior, task quality, component health, and maintenance decisions.

The sensing stage provides the measurements on which all subsequent decisions depend. Cameras, vibration sensors, GNSS/IMU units, load sensors, and machine-state sensors provide information about the crop, terrain, machine configuration, and component condition. Sensor fusion combines complementary measurements to support the state estimates used throughout this chain. Sensor noise, electromagnetic interference, mounting variation, calibration error, temporal misalignment, signal drift, and data loss can compromise individual measurements and the resulting fused estimates. These errors may then affect perception outputs, such as crop boundaries, machine pose, header height, blockage probability, or component-health indicators, and propagate into subsequent decisions. A high perception accuracy measured under controlled conditions does not guarantee a reliable control decision if the perception output is delayed, poorly synchronized, or accompanied by unreported uncertainty.

At the decision stage, the estimated state is converted into an operational objective, such as a path, forward speed, feed rate, header height, or maintenance priority. If uncertainty, timestamp differences, or sensor-health information are ignored, the decision module may generate commands that are locally reasonable but unsuitable for the current machine state. The control module must then compensate for disturbances, model mismatch, actuator delay, and changing soil or crop conditions. Errors that are not corrected at this stage can appear as path deviation, header-height error, speed oscillation, excessive actuator effort, crop loss, or increased mechanical loading.

A representative example is the control of a combine harvester operating under changing terrain and feed conditions. Multi-sensor measurements are first used to estimate machine attitude, terrain variation, header height, and internal operating state. These estimates support decisions concerning travel speed, header position, and feeding regulation. If vibration, signal delay, or sensor drift causes the estimated header height or machine attitude to deviate from the actual state, the controller may issue an inappropriate hydraulic or propulsion command. The resulting actuation error can increase header deviation, grain loss, impurity content, energy consumption, or structural loading. Residual vibration and load signals generated during operation can subsequently be used for blockage detection, fault diagnosis, or structural-health assessment. The maintenance decision should therefore consider not only the diagnostic probability but also the reliability of the upstream sensing and control information.

This chain shows why module-level metrics should be connected to task-level and maintenance-level outcomes. Perception studies should report uncertainty, latency, synchronization, and sensor-degradation sensitivity; control studies should report actuator effort, stability margins, and performance under delayed or corrupted measurements; and maintenance studies should report false alarms, missed faults, remaining-useful-life uncertainty, downtime, and repair consequences. End-to-end validation should trace how a specified sensing disturbance propagates through the decision and control layers and changes task quality, component health, and maintenance action. Comparisons between fused inputs and individual-sensor inputs under matched operating and evaluation conditions should assess whether fusion reduces downstream errors and improves fault detection and maintenance decisions. At present, most studies provide only partial evidence for this chain, and complete machine-level error-propagation experiments remain an important research priority. Addressing these system-level weaknesses will require a shift from module-level validation toward maintenance strategies that link machine condition to operational and economic decisions.

### 8.4. Future Directions for Decision-Oriented Intelligent Maintenance

Because current evidence remains concentrated on component-level fault recognition and health assessment, future research should extend intelligent O&M toward decision-making across the machine lifecycle. Existing studies largely emphasize classification accuracy, fault-detection rates, or health-assessment performance, while giving limited attention to how these outputs influence maintenance timing, machine availability, repair coordination, and operating costs. A practical framework should therefore connect sensing and health assessment with degradation forecasting, maintenance planning, field repair, and post-maintenance verification.

Remaining useful-life (RUL) prediction is central to this transition. Fault diagnosis indicates whether an abnormal condition is present, whereas RUL estimation concerns how that condition will develop before functional failure. This task is complicated by changes in load, soil resistance, crop material, operating speed, and seasonal conditions. RUL models should report prediction uncertainty, warning lead time, degradation trajectories, and robustness to changing environments. Their performance should also be assessed across machines and seasons, including situations involving partial sensor failure, rather than being validated only on controlled laboratory degradation data.

Health estimates become valuable only when they support an operational decision. Condition-based maintenance systems should help determine whether a machine can continue operating, whether it should enter a degraded mode, or whether inspection and repair are required. Such decisions depend on more than the estimated probability of failure. They also involve the consequences of failure, the remaining workload, the availability of replacement machines, and the narrow seasonal windows in which agricultural operations must be completed. This calls for risk-based intervention policies that account for operational context instead of relying on fixed alarm thresholds.

Reliable decision-making requires explicit monitoring of sensor health. Vibration, pressure, temperature, current, position, and load sensors can be affected by drift, loose mounting, saturation, electromagnetic interference, intermittent data loss, or calibration changes. A system may produce a confident but incorrect diagnosis when the sensing layer has deteriorated. Sensor assessment should incorporate signal plausibility, agreement among related measurements, temporal continuity, calibration status, and confidence estimates. When sensor reliability declines, the system should be able to draw on redundant measurements and operate in a degraded mode.

Maintenance planning must further balance competing risks and costs. Immediate replacement can reduce the chance of failure but may create unnecessary downtime and expenditure, whereas delayed intervention can lead to secondary damage, production losses, or unsafe operation. Decision models should therefore consider failure probability together with maintenance cost, downtime, repair duration, spare-parts availability, and task quality. Their evaluation should extend beyond diagnostic metrics to include avoided downtime, unnecessary-maintenance rate, the cost of missed faults, emergency-repair frequency, and machine availability.

Field conditions make this problem more demanding. AM often operates across dispersed locations where spare parts, service vehicles, and trained personnel are not readily available. A useful maintenance system should estimate where service is likely to be required, which parts and tools will be needed, how long the repair may take, and whether temporary degraded operation is feasible. Linking health prediction with inventory management, field location, service routing, and operator availability could shorten repair delays and improve productivity during critical seasonal periods.

The economic value of intelligent maintenance cannot be established through diagnostic accuracy alone. Lifecycle assessment should include sensing and computing hardware, energy use, inspection frequency, labor, spare parts, downtime, component replacement, retrofit requirements, and residual machine value. Economic comparisons should assess whether the system reduces operating cost per hour, extends component life, and increases machine availability. Any payback estimate should state its operating assumptions clearly and distinguish measured outcomes from prospective targets.

Taken together, these directions define a broader evaluation framework for intelligent AM. Computational feasibility, cross-scenario robustness, system integration, and safety assurance should ultimately be evaluated according to their effects on machine-level reliability and maintenance decisions. Future studies should trace an evidence chain from sensor integrity and machine condition to degradation prediction, intervention planning, repair execution, and lifecycle outcomes. The present literature does not yet provide sufficient evidence to support routine farm-level deployment of such integrated maintenance systems, but establishing this evidence chain is essential for demonstrating sustained benefits at both the whole-machine and farm-management levels.

### 8.5. Limitations of the Review

An important limitation of this review is the uneven distribution of the systematically charted evidence across the four technical domains. Among the 147 charted evidence sources, 69 addressed agricultural machinery automation, 40 focused on intelligent perception, 15 addressed dynamic modeling and mechanical-system design, and only three were assigned primarily to intelligent O&M. Although the supplementary contextual search and the expanded discussion in Section 7 and Section 8.4 broaden the coverage of O&M-related topics, these materials do not change the composition of the systematically charted evidence base. Consequently, conclusions concerning intelligent O&M are supported by a smaller and less comprehensive body of directly charted evidence than conclusions concerning automation, perception, and mechanical-system design. The O&M discussion should therefore be interpreted as a synthesis of an emerging evidence base and an identification of research priorities, rather than as a comprehensive assessment of the maturity or effectiveness of intelligent maintenance in agricultural machinery. Future systematic studies should expand the field-validated evidence on condition monitoring, remaining-useful-life prediction, maintenance scheduling, sensor-health assessment, repair logistics, and lifecycle outcomes.

## 9. Conclusions

This review synthesizes AI applications in AM from 2014 to early 2026, examining how sensing and information fusion connect perception, operational control, condition monitoring, and fault diagnosis. Mechanical modeling provides context for interpreting operating responses, while perception methods extract features describing crops, field conditions, and machine states. Control systems translate these estimates into operational actions. Within this broader chain, blockage detection and multi-component monitoring illustrate how complementary measurements can support abnormal-state identification and intervention. The contribution of fusion therefore depends on the information supplied by each input and the decision that the combined information supports.

The reviewed evidence also reveals important distinctions between improved measurement, operational performance, and diagnostic capability. Advances in target recognition, navigation, or control provide enabling technologies, but do not independently establish improved fault diagnosis. Diagnostic benefits require comparisons that isolate the contribution of complementary inputs under relevant operating conditions. Noise, changing loads, calibration errors, temporal misalignment, and sensor degradation can obscure useful features or introduce inconsistencies into fused estimates. Evidence remains limited for sustained diagnostic performance across machines and seasons, particularly for early warning and precise fault localization under degraded sensing.

Overcoming this laboratory-to-field gap requires coordinated sensing, computation, and validation under representative operating conditions. Fusion systems should account for measurement timing, calibration, and input reliability when combining information, with fallback behavior defined for degraded sensing or communication. To reconcile computational demands with field power constraints, neural networks should be optimized to meet the power and computational limits of the deployment hardware. Evaluation should consider power consumption and execution latency in relation to the requirements of multimodal perception, motion control, and tactile gripping. Shared datasets and interfaces should preserve the operating conditions and sensor-quality information needed to interpret results and trace errors from acquisition to downstream decisions.

Moving forward, future progress must be evaluated by verifiable, system-level targets rather than solitary statistical improvements. Comparisons between fused and individual inputs should assess performance under changing loads, noise, and partial sensor failure. Where early warning or fault localization is claimed, evaluation should report warning lead time and localization performance alongside diagnostic errors. System safety should be assessed using fault detection rates, false-alarm rates, fallback response times during communication loss, and autonomous recovery performance. Acceptable response times should be determined by the operational risks and safety requirements of the specific task. Practical value also depends on deployment and maintenance demands. Relevant considerations include equipment retrofit costs, operator skill requirements, service downtime, and commercial payback periods. Economic viability should be assessed in relation to farm scale, equipment utilization, and operation and maintenance costs. Ultimately, the value of sensor fusion should be established through reliable state information that supports fault detection, timely intervention, and informed maintenance decisions under field conditions.

## Figures and Tables

**Figure 1:**
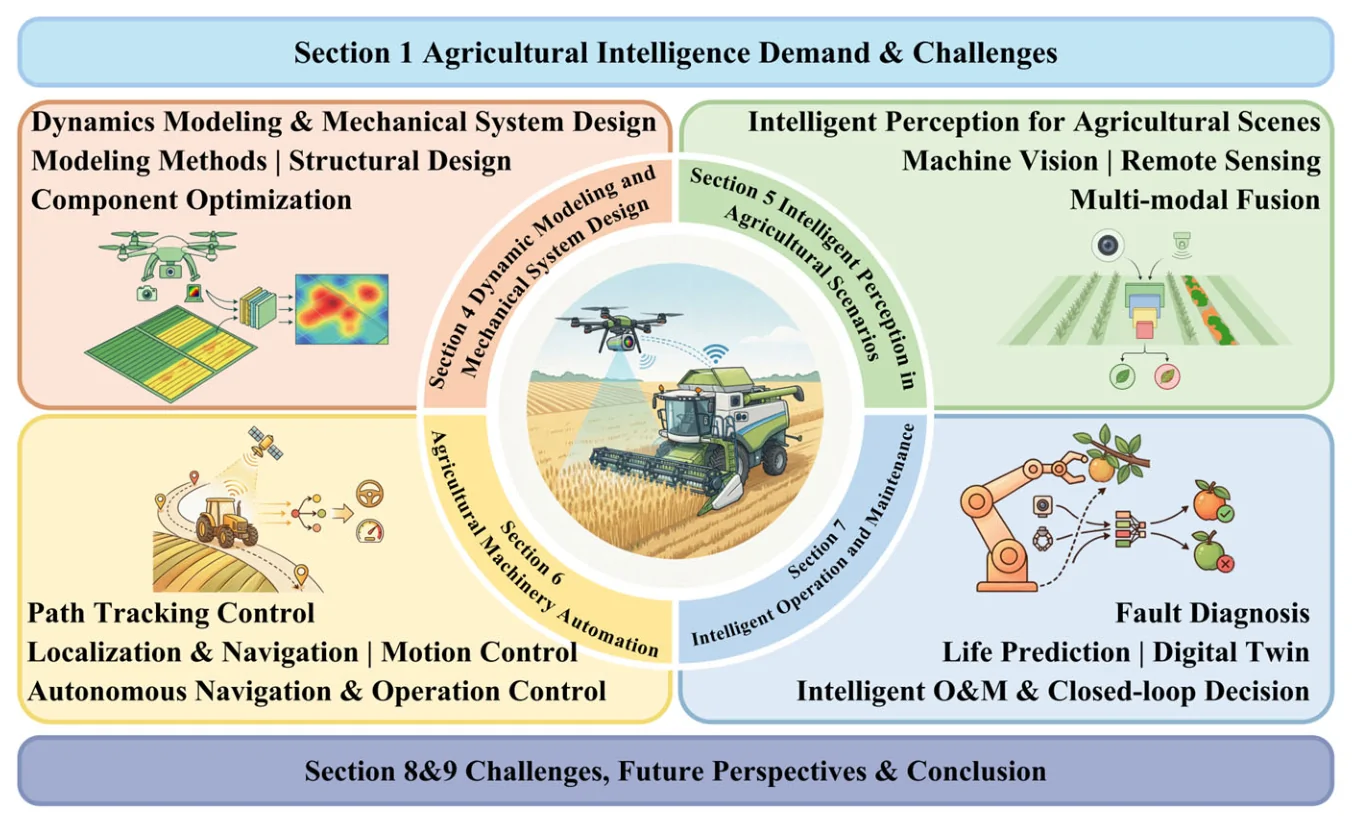
Framework of the review.

**Figure 2:**
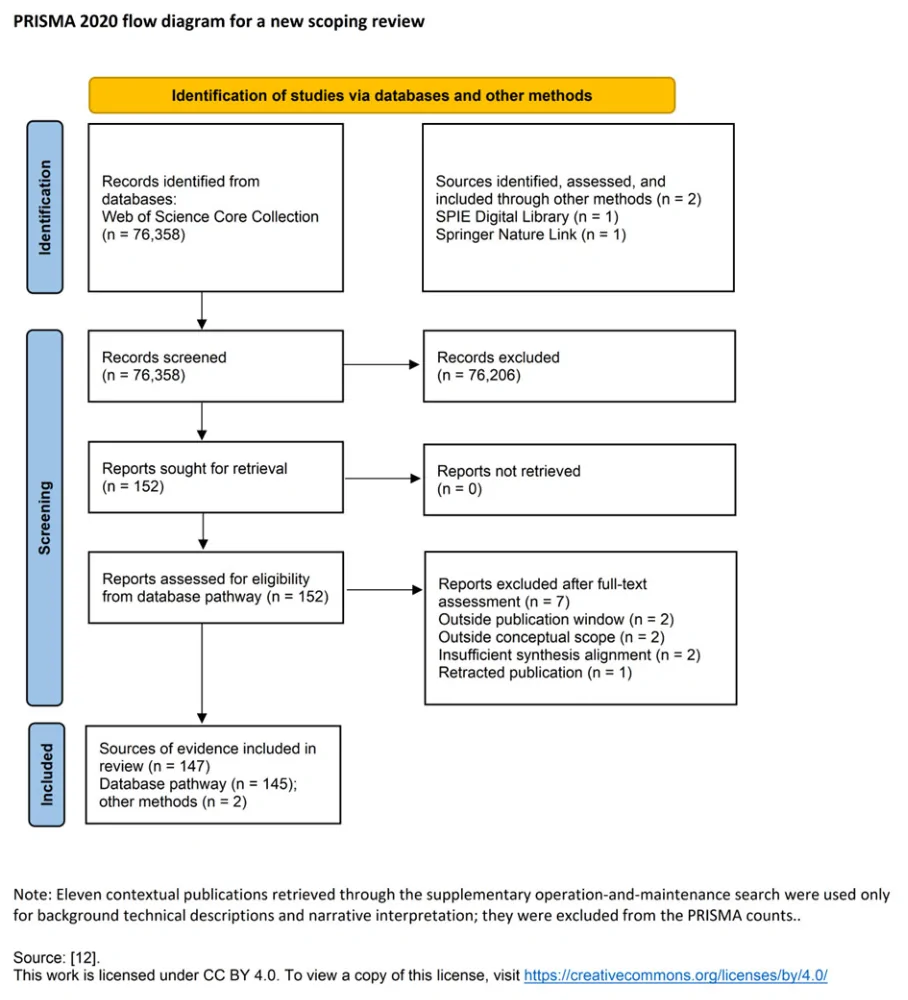
PRISMA 2020 flow diagram of the study-selection process, including database and other-method sources.

**Figure 3:**
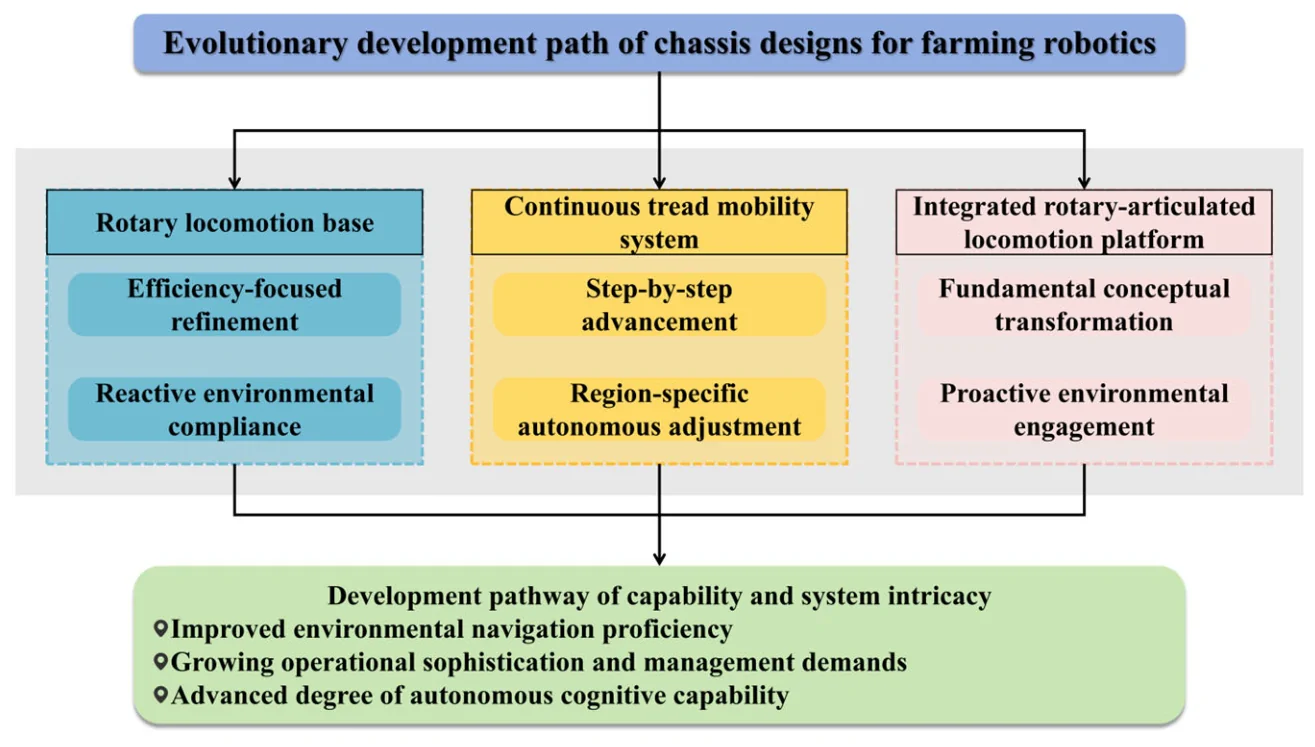
Chassis evolution in agricultural robotics [18].

**Figure 4:**
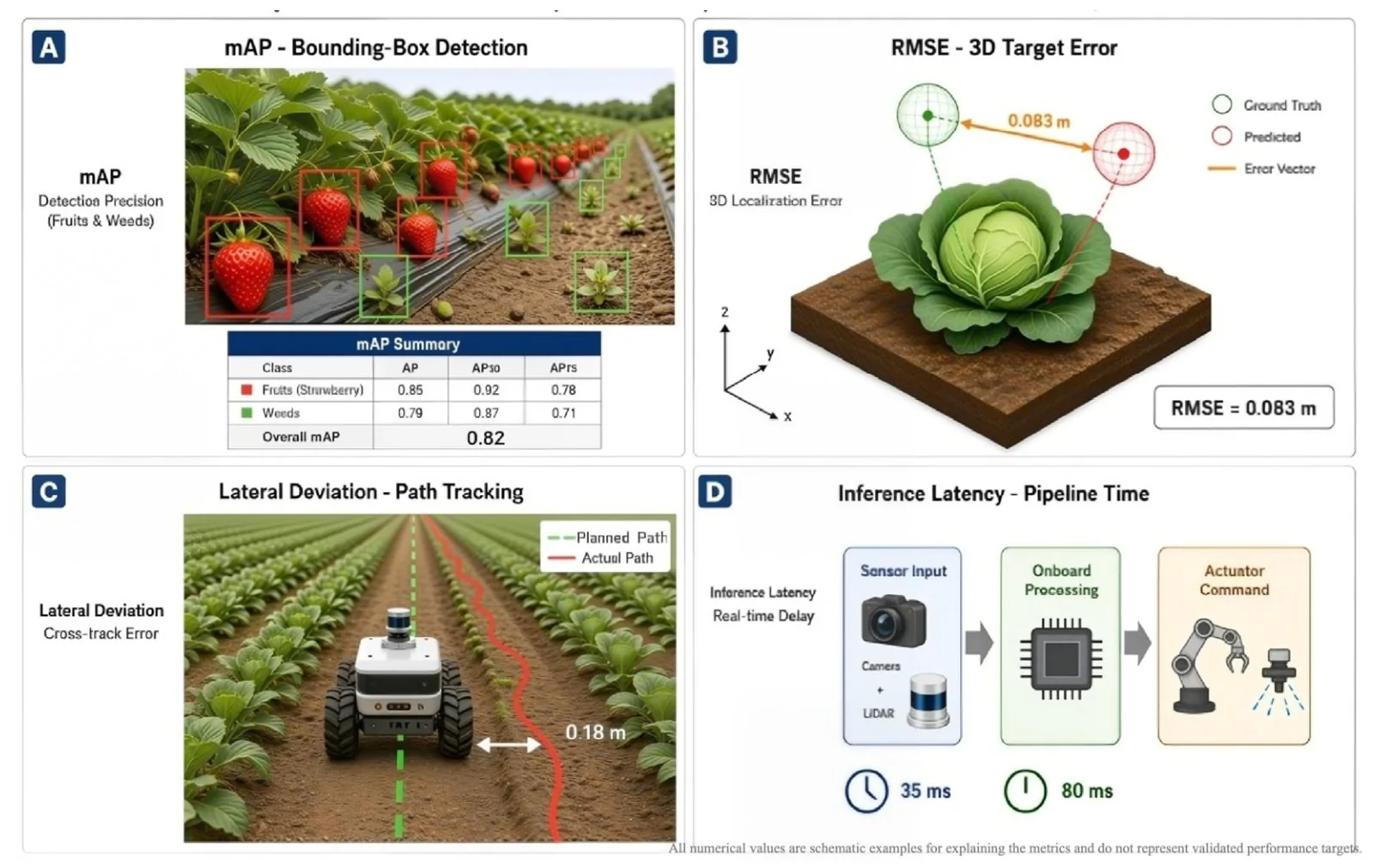
Methodological metrics and their operational implications in AM validation. Note: The numerical values shown in panels (**A**–**D**) are illustrative examples for explaining the metrics; they are not experimental measurements, literature-derived data, or validated performance targets.

**Figure 5:**
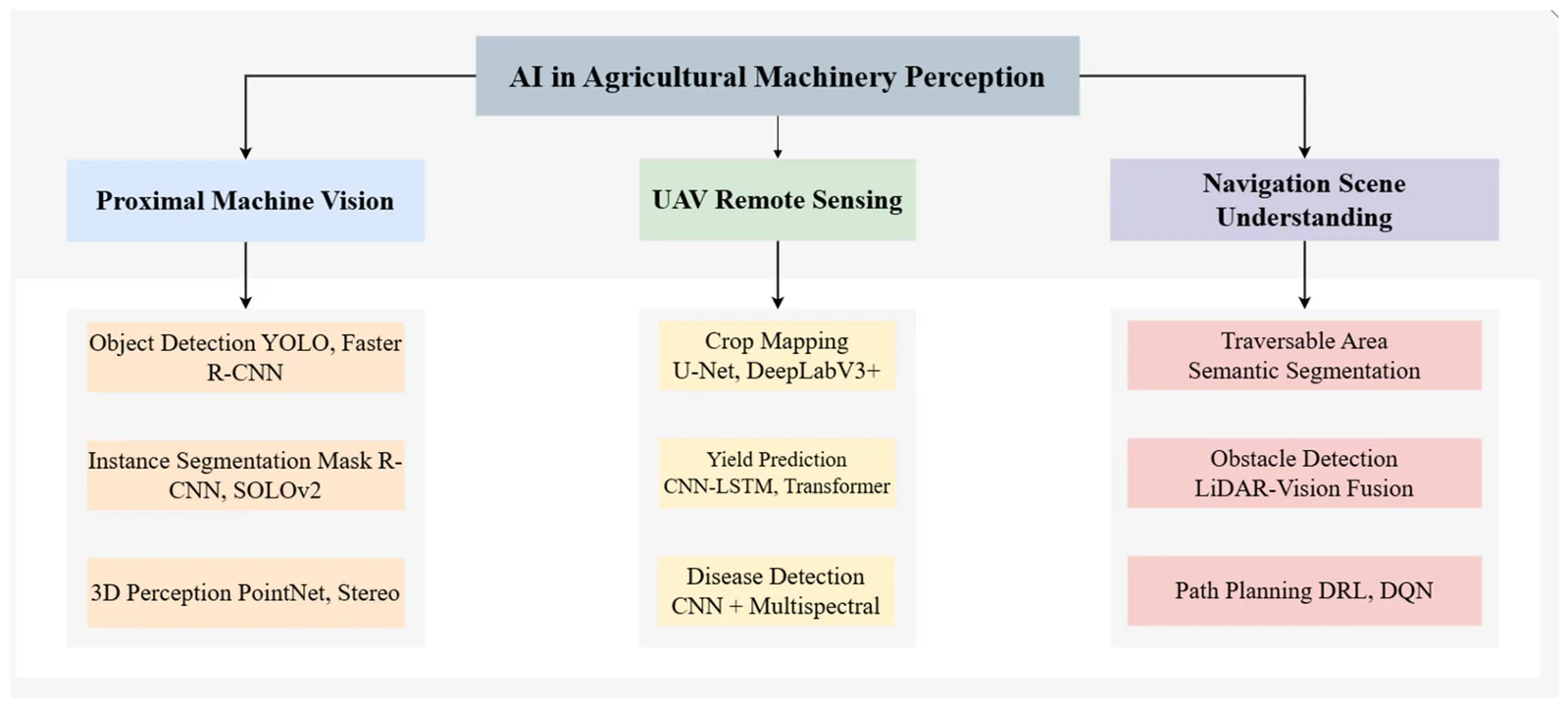
Classification framework of AI-based perception technologies for AM.

**Figure 6:**
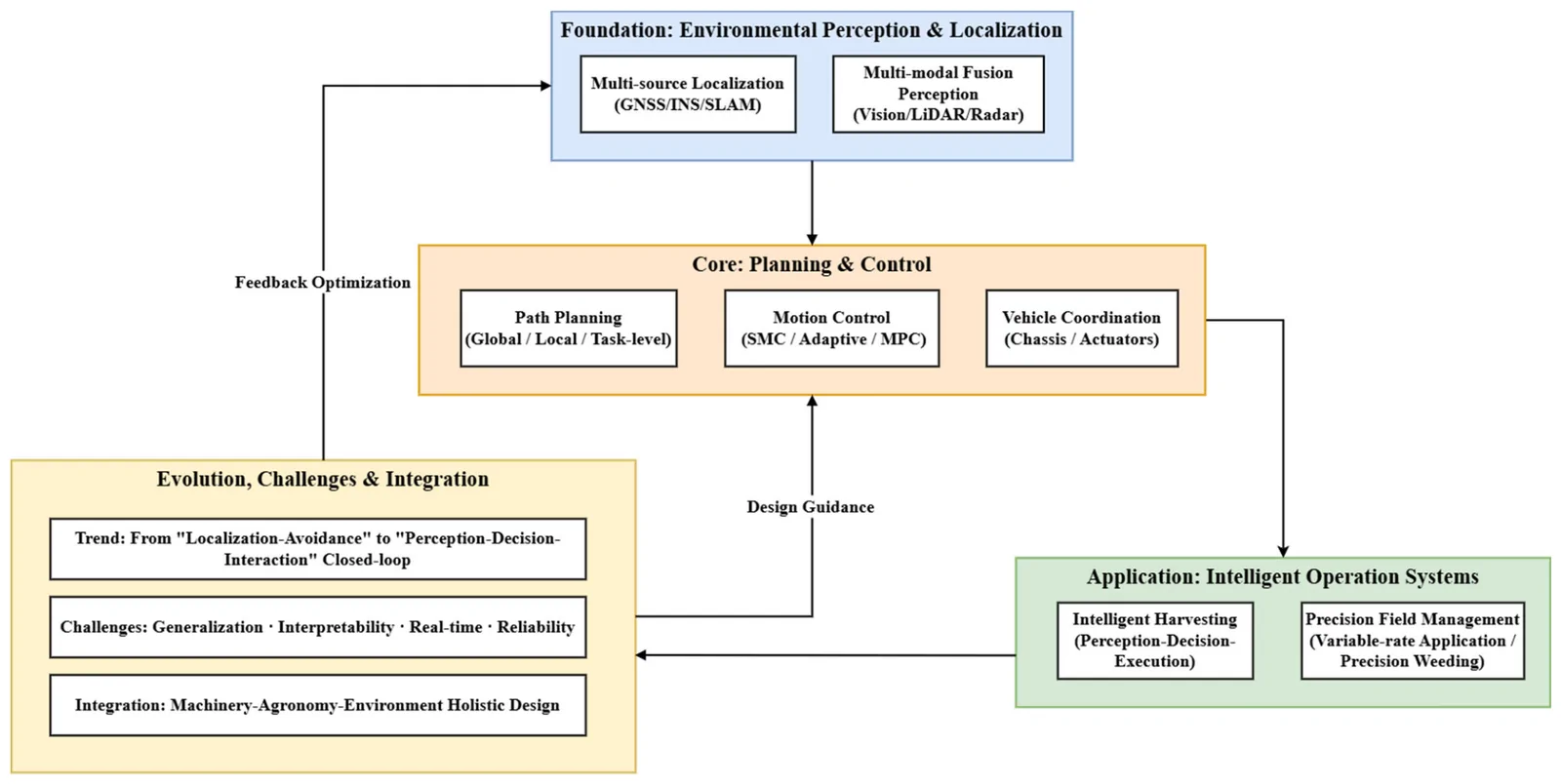
Architecture of autonomous AM systems.

**Figure 7:**
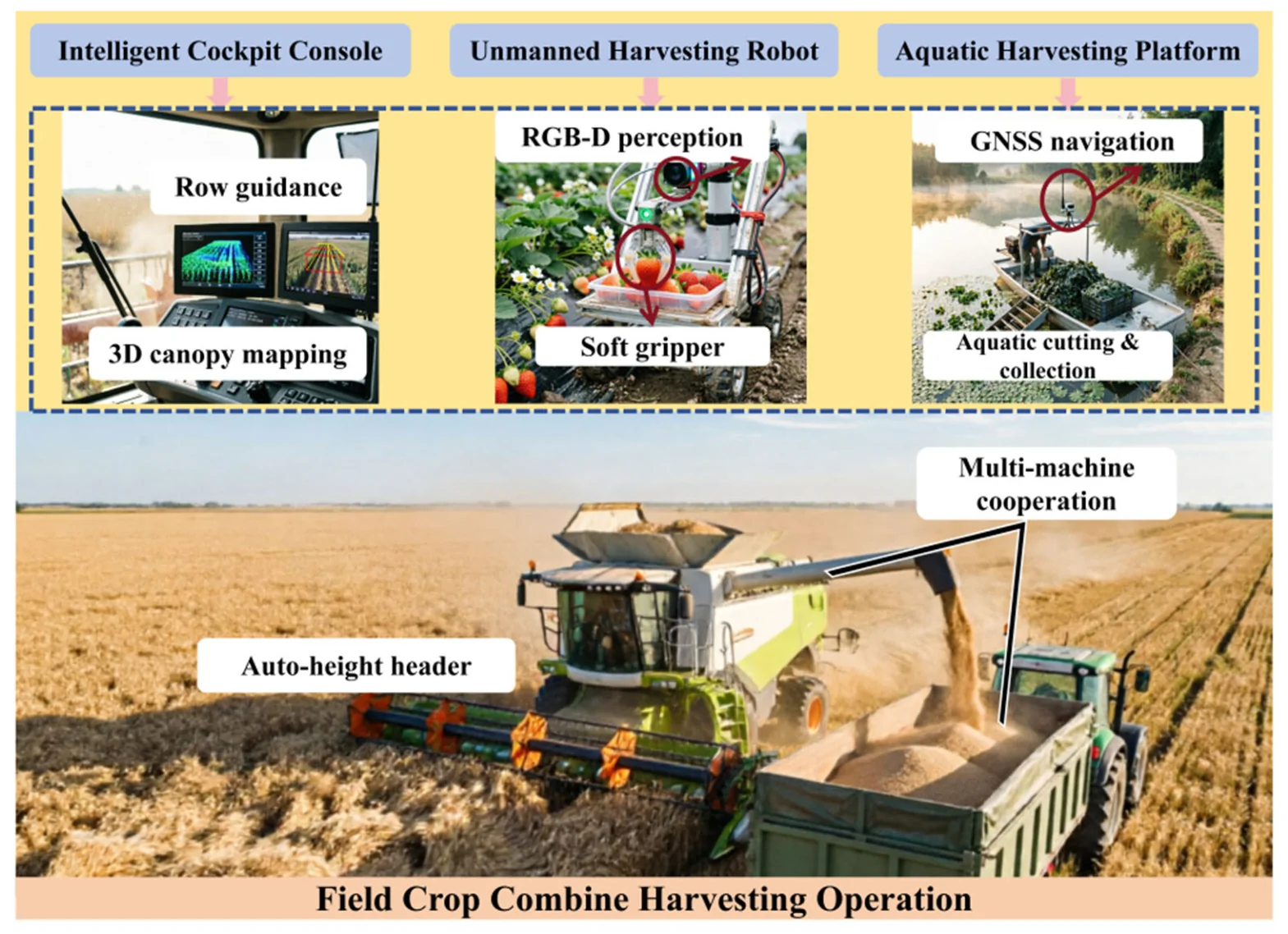
Intelligent harvesting: from perception to adaptive actuation.

**Table 1:** Environmental variability and sensing constraints in agricultural field operations.

Dimensions	Core Manifestations
Spatial Heterogeneity	Terrain, soil properties, and crop distribution vary significantly at the meter scale.
Temporal Dynamicity	Illumination, temperature-humidity conditions, and crop morphology fluctuate across hourly to seasonal timescales.
Semantic Ambiguity	Crops closely resemble the background in color and texture, and target boundaries shift continuously across growth stages.
Physical Interaction Complexity	Mechanical coupling between the machine, soil, and crops is influenced by multiple factors such as moisture content and root systems.
Sensing and Information-Acquisition Constraints	Dust, canopy occlusion, and electromagnetic interference degrade sensor signal quality.

**Table 2:** Representative approaches to data-driven modeling and the incorporation of physical information in AM.

Approach	Main Function	Relevance to AM	Main Limitation
Pure data-driven learning	Learn input-output mapping from images, signals, or operation logs.	Fruit detection, weed identification, fault classification with sufficient data.	Weak extrapolation under unseen crops, soil, illumination, or sensor drift.
Physics-informed neural network (PINN)	Embed governing equations, boundary conditions, or physical residuals into learning.	Soil-tool interaction, traction-slip estimation, airflow/vibration modeling.	Requires credible physical equations and careful loss balancing.
Simulation-assisted surrogate model	Use DEM/CFD/FEM/multibody simulation to train fast neural surrogates.	Harvester cleaning, chassis vibration, fatigue and load prediction.	Simulation-to-field gap remains if calibration data are limited.
Digital twin with data assimilation	Continuously update a virtual machine model using sensor feedback.	Predictive maintenance and whole-machine health assessment.	High model-building cost and real-time synchronization difficulty.
Constraint-based safety layer	Place physical and agronomic limits around data-driven control outputs.	Navigation, adaptive speed control, end-effector force regulation.	May reduce performance if constraints are overly conservative.

**Table 3:** Scenario-level validation and deployment characteristics of representative perception algorithms for AM.

Scenario	Representative Methods	Examples of Validation Settings	Evaluation Metrics	Recommended Reporting Requirement
Fruit/vegetable target detection	SVM, CNN, YOLO, Mask R-CNN, transformer-based detectors	Fruit and vegetable detection under occlusion, background clutter, and variable illumination.	mAP, recall, recognition rate, inference latency	Report dataset composition, evaluation splits, and computing hardware. Distinguish original images from augmented samples and assess performance across relevant operating conditions.
Navigation obstacle detection	YOLO variants, lightweight CNNs, attention-enhanced detectors	Obstacle detection in orchard and other navigation scenes with vegetation interference and changing illumination.	Precision, recall, false-positive rate, false-negative rate, end-to-end latency	State model size, target hardware, and operating speed. Report missed obstacles and false alarms, together with their effects on navigation.
Crop-row or boundary segmentation	U-Net, DeepLabV3+, multitask detection–segmentation networks	Crop-row and field-boundary extraction under weed interference, discontinuous rows, and variable lighting.	Intersection over union, boundary error, processing latency; downstream lateral tracking error	Define how boundary error is measured and connect segmentation results to path tracking or cutting/spraying quality. Distinguish image-level metrics from machine-level outcomes.
3D geometric perception	LiDAR point-cloud processing, PointNet-style models, attention-based point-cloud networks	Orchard inter-row localization and crown–trunk separation using geometric measurements.	3D localization error, task-specific classification or segmentation metrics, processing latency	Describe sensor placement, point-cloud density, and reference measurements. Identify whether the method uses a single sensing modality or combines additional inputs.
Multimodal perception and localization	RGB-D perception, image–point-cloud integration, multimodal pose-estimation networks	Target localization and scene interpretation using registered appearance and depth or geometric information.	3D localization error, pose-estimation error, end-to-end latency; performance under degraded or missing inputs	Identify input modalities and the fusion stage. Report calibration and synchronization procedures, and compare fused and individual inputs under matched conditions.

**Table 4:** Strengths, limitations, and practical applicability of main AI technologies in AM.

AI Technique	Main Use in AM	Strengths	Limitations	Best Suited Scenario
CNN/YOLO-family detectors	Object detection, crop-row recognition, obstacle detection.	Mature visual feature extraction; many lightweight variants.	Sensitive to unseen illumination, crop stage, occlusion, and camera viewpoint.	Real-time visual perception with annotated image data.
Transformer/attention models	Occluded target detection and multimodal fusion.	Captures long-range contextual features.	Computationally heavier and data-hungry.	Complex scenes with severe occlusion or weak contrast.
Classical machine learning	Small-sample sensing and fault classification.	Works with limited data and handcrafted features.	Weak robustness to high-dimensional visual variability.	Low-data diagnostic tasks.
Reinforcement learning	Path planning, task allocation, adaptive operation policies.	Optimizes sequential decisions.	Training and safety transfer remain difficult.	Simulation-rich planning before field transfer.
Physics-informed learning	Soil-tool, traction-slip, airflow/vibration, safe control.	Constrains outputs using mechanistic knowledge.	Requires reliable physical relationships.	Mechanically constrained AM problems.
Digital twins	Health assessment and virtual safety testing.	Links sensing, simulation, diagnosis, and decision support.	High modeling and synchronization cost.	High-value machines with maintenance planning needs.

**Table 5:** Stage-wise information flow and error propagation from sensing to maintenance decision-making in AM.

Stage	Typical Input	Stage Output	Main Error Source	Propagated Consequence	Engineering Indicators
Sensing	Vibration, images, position, inertial and load signals	Raw sensor measurements	Noise, drift, missing data, calibration error, electromagnetic interference	Biased or incomplete state estimation	Signal-to-noise ratio, missing-data rate, calibration error
Perception	Sensor measurements and synchronized multisensor data	Crop boundary, machine state, fault probability	Occlusion, desynchronization, domain shift	Biased or unreliable decision input	Detection error, localization error, confidence calibration
Decision	Estimated state, associated uncertainty and task objectives	Target speed, planned path, feed-rate target or maintenance priority	Ignored uncertainty, outdated state estimates, inappropriate decision rules	Unsafe or inefficient operational decisions	Decision latency, constraint-violation rate, false-alarm rate
Control	Reference commands and measured machine state	Actuator control signals	Model mismatch, feedback delay, unaccounted actuator limits	Tracking deviation, oscillation or excessive control effort	Tracking error, response time, control effort
Actuation	Control signals and mechanical loads	Physical motion and task execution	Wear, backlash, slip, saturation, blockage	Reduced task quality or increased component stress	Actuation error, crop loss, energy consumption
Monitoring and maintenance	Condition signals, control residuals and operating history	Health estimate, remaining useful-life (RUL) estimate and maintenance action	Incomplete degradation data, model mismatch, prediction uncertainty	Delayed repair, unnecessary maintenance or excessive downtime	False-negative rate, RUL prediction error, downtime, lifecycle cost

## Data Availability

The raw data supporting the conclusions of this article will be made available by the authors on request.

## Supplementary Materials

The following supporting information can be downloaded at: https://www.mdpi.com/article/10.3390/s26196029/s1. Table S1: Bibliographic and charted characteristics of the 147 included sources of evidence. Table S1 contains 145 sources verified in the Web of Science export and two conference sources identified through other methods. The eleven additional contextual sources are listed in Table S3. NR, not reported. Table S2: Publications excluded after full-text assessment and their primary reasons. Each publication is assigned one primary reason in accordance with the eligibility and synthesis framework. Table S3: Primary search, other-method sources, and eleven contextual publications. Table S4: Structured evidence table for the 147 included sources. “Not reported in source” indicates that the information was not explicitly stated or could not be verified. “Not applicable (review study)” indicates that the source was a review without a single primary dataset or experimental validation setting. Performance values are study-specific and should not be directly compared across heterogeneous machinery, crops, datasets, hardware platforms, operating conditions, and validation protocols.

## References

[1] Jiang L., Xu B., Husnain N., Wang Q. (2025). Overview of Agricultural Machinery Automation Technology for Sustainable Agriculture. Agronomy.

[2] Ammar E.E., Aziz S.A., Zou X., Elmasry S.A., Ghosh S., Khalaf B.M., EL-Shershaby N.A., Tourky G.F., AL-Farga A., Khan A.N. (2024). An In-Depth Review on the Concept of Digital Farming. Environ. Dev. Sustain..

[3] Zhu Y., Zhang S., Tang S., Gao Q. (2025). Research Progress and Applications of Artificial Intelligence in Agricultural Equipment. Agriculture.

[4] Kuan Y.N., Goh K.M., Lim L.L. (2025). Systematic Review on Machine Learning and Computer Vision in Precision Agriculture: Applications, Trends, and Emerging Techniques. Eng. Appl. Artif. Intell..

[5] Zhao J., Fan S., Zhang B., Wang A., Zhang L., Zhu Q. (2025). Research Status and Development Trends of Deep Reinforcement Learning in the Intelligent Transformation of Agricultural Machinery. Agriculture.

[6] Ge C., Zhang G., Wang Y., Shao D., Song X., Wang Z. (2025). Research Status and Development Trends of Artificial Intelligence in Smart Agriculture. Agriculture.

[7] Shen Y., Yang F., Wu J., Luo S., Khan Z., Zhang L., Liu H. (2025). Advances and Future Trends in Electrified Agricultural Machinery for Sustainable Agriculture. Agriculture.

[8] Qin S., Zhang S., Zhong W., He Z. (2025). Control Algorithms for Intelligent Agriculture: Applications, Challenges, and Future Directions. Processes.

[9] Jin Y., Liu J., Xu Z., Yuan S., Li P., Wang J. (2021). Development Status and Trend of Agricultural Robot Technology. Int. J. Agric. Biol. Eng..

[10] Liu Q., Yu R., Suo H., Cai Y., Chen L., Jiang H. (2025). Autonomous Driving in Agricultural Machinery: Advancing the Frontier of Precision Agriculture. Actuators.

[11] Tricco A.C., Lillie E., Zarin W., O’Brien K.K., Colquhoun H., Levac D., Moher D., Peters M.D.J., Horsley T., Weeks L. (2018). PRISMA Extension for Scoping Reviews (PRISMA-ScR): Checklist and Explanation. Ann. Intern. Med..

[12] Page M.J., McKenzie J.E., Bossuyt P.M., Boutron I., Hoffmann T.C., Mulrow C.D., Shamseer L., Tetzlaff J.M., Akl E.A., Brennan S.E. (2021). The PRISMA 2020 Statement: An Updated Guideline for Reporting Systematic Reviews. BMJ.

[13] Jiang Z., Chang Y., Shehzad A., Lei R., Sun W., Hu X., Cheng G. (2026). Dynamics and Vibration Analysis of Agricultural Machinery Systems: Recent Advances and Future Perspectives. J. Braz. Soc. Mech. Sci. Eng..

[14] Tang S., Gu J., Tang K., Ding W., Shang Z. (2019). Eigen Solution of Neural Networks and Its Application in Prediction and Analysis of Controller Parameters of Grinding Robot in Complex Environments. Complexity.

[15] Ma Z., Zhu Y., Wu Z., Nfamoussa Traore S., Chen D., Xing L. (2023). BP Neural Network Model for Material Distribution Prediction Based on Variable Amplitude Anti-Blocking Screening DEM Simulations. Int. J. Agric. Biol. Eng..

[16] Li Q., Wan R., Wu Z., Yan Y., Zhang X. (2025). Intelligent Analysis of Flow Field in Cleaning Chamber for Combine Harvester Based on YOLOv8 and Reasoning Mechanism. Appl. Sci..

[17] Ding R., Qi X., Chen X., Mei Y., Li A. (2025). The Current Development Status of Agricultural Machinery Chassis in Hilly and Mountainous Regions. Appl. Sci..

[18] Ding R., Qi X., Meng X., Chen X., Zhang L., Mei Y., Li A., Ye Q. (2025). A Review on the Chassis Configurations and Key Technologies of Agricultural Robots. Agriculture.

[19] Jiang Y., Wang R., Ding R., Sun Z., Jiang Y., Liu W. (2025). Research Review of Agricultural Machinery Power Chassis in Hilly and Mountainous Areas. Agriculture.

[20] Jiang Y., Sun Z., Wang R., Ding R., Ye Q. (2024). Design and Control of a New Omnidirectional Levelling System for Hilly Crawler Work Machines. Comput. Electron. Agric..

[21] Gao W., Liu J., Deng J., Jiang Y., Jin Y. (2025). Research Status and Trends in Universal Robotic Picking End-Effectors for Various Fruits. Agronomy.

[22] Yang Q., Qu G., Zhong X., Yang X., Liu L., Hu X., Addy M.M. (2026). Design and Performance Evaluation of a Rigid-Flexible Coupling End-Effector for Tomato Picking Robots. Front. Agric. Sci. Eng..

[23] Zhang F., Chen Z., Wang Y., Bao R., Chen X., Fu S., Tian M., Zhang Y. (2023). Research on Flexible End-Effectors with Humanoid Grasp Function for Small Spherical Fruit Picking. Agriculture.

[24] Lu Y., Xu W., Leng J., Liu X., Xu H., Ding H., Zhou J., Cui L. (2024). Review and Research Prospects on Additive Manufacturing Technology for Agricultural Manufacturing. Agriculture.

[25] Wang S., Li B., Chen S., Tang Z., Zhou W., Guo X. (2024). Design and Performance Test of Soybean Profiling Header Suitable for Harvesting Bottom Pods on Film. Agriculture.

[26] Cui L., Mao H., Xue X., Ding S., Qiao B. (2018). Design Optimization and Test for a Pendulum Suspension of the Crop Sprayer Boom in Dynamic Conditions Based on a Six DOF Motion Simulator. Int. J. Agric. Biol. Eng..

[27] Liang Z., Jiang Q. (2026). A Deep Learning-Enhanced Low-Cost Cylindrical Anemometer for Combine Harvester Cleaning Systems. Comput. Electron. Agric..

[28] Lü Q., Cai J., Liu B., Deng L., Zhang Y. (2014). Identification of Fruit and Branch in Natural Scenes for Citrus Harvesting Robot Using Machine Vision and Support Vector Machine. Int. J. Agric. Biol. Eng..

[29] Jia W., Mou S., Wang J., Liu X., Zheng Y., Lian J., Zhao D. (2020). Fruit Recognition Based on Pulse Coupled Neural Network and Genetic Elman Algorithm Application in Apple Harvesting Robot. Int. J. Adv. Robot. Syst..

[30] Tai S., Tang Z., Li B., Wang S., Guo X. (2025). Intelligent Recognition and Automated Production of Chili Peppers: A Review Addressing Varietal Diversity and Technological Requirements. Agriculture.

[31] Ji W., Gao X., Xu B., Pan Y., Zhang Z., Zhao D. (2021). Apple Target Recognition Method in Complex Environment Based on Improved YOLOv4. J. Food Process Eng..

[32] Ji W., Zhai K., Xu B., Wu J. (2025). Green Apple Detection Method Based on Multidimensional Feature Extraction Network Model and Transformer Module. J. Food Prot..

[33] Tang S., Xia Z., Gu J., Wang W., Huang Z., Zhang W. (2024). High-Precision Apple Recognition and Localization Method Based on RGB-D and Improved SOLOv2 Instance Segmentation. Front. Sustain. Food Syst..

[34] Guo C., Zhu C., Liu Y., Huang R., Cao B., Zhu Q., Zhang R., Zhang B. (2024). End-to-End Lightweight Transformer-Based Neural Network for Grasp Detection towards Fruit Robotic Handling. Comput. Electron. Agric..

[35] Chen J., Fu S., Wang Z., Zhu L., Xia H., Wu F., Cen F. (2021). Research on the Method of Predicting Feeding Volume of Rice Combine Harvester Base on Machine Vision. Proceedings of the International Conference on Image Processing and Intelligent Control (IPIC 2021), Lanzhou, China, 30 July–1 August 2021.

[36] Zhang Q., Chen Q., Xu W., Xu L., Lu E. (2024). Prediction of Feed Quantity for Wheat Combine Harvester Based on Improved YOLOv5s and Weight of Single Wheat Plant without Stubble. Agriculture.

[37] Zhang Q., Hu J., Xu L., Cai Q., Yu X., Liu P. (2023). Impurity/Breakage Assessment of Vehicle-Mounted Dynamic Rice Grain Flow on Combine Harvester Based on Improved Deeplabv3+ and YOLOv4. IEEE Access.

[38] Liu P., Cui Z., Hu J., Zhang Q., Sun J., Chai X., Xu L. (2025). Lodge-Unet: A Dual-Frequency Feature Fusion Network with Boundary-Aware Optimization for Wheat Lodging Detection via Autonomous Harvesters. Comput. Electron. Agric..

[39] Xu J., Liu H., Shen Y. (2025). Image and Point Cloud-Based Neural Network Models and Applications in Agricultural Nursery Plant Protection Tasks. Agronomy.

[40] Zhang M., Zhang B., Zhao C., Chen L., Kuai Y., Wang C., Jiang S., Chen D., Zhu Q., Wang Z. (2025). Tobacco Yield Estimation via Multi-Source Data Fusion and Recurrent Neural Networks. Int. J. Appl. Earth Obs. Geoinf..

[41] Wei L., Yang H., Niu Y., Zhang Y., Xu L., Chai X. (2023). Wheat Biomass, Yield, and Straw-Grain Ratio Estimation from Multi-Temporal UAV-Based RGB and Multispectral Images. Biosyst. Eng..

[42] Zhao J., Li H., Liu J., Zhao M., Yan Z. (2025). Integration of Spatial Attention Mechanism into 1D-CNN for Prediction of Wheat Leaf Nitrogen Concentration from UAV-Borne Hyperspectral Imagery. Comput. Electron. Agric..

[43] Niu Y., Song X., Zhang L., Xu L., Wang A., Zhu Q. (2025). Enhancing Model Accuracy of UAV-Based Biomass Estimation by Evaluating Effects of Image Resolution and Texture Feature Extraction Strategy. IEEE J. Sel. Top. Appl. Earth Obs. Remote Sens..

[44] Ma W., Han W., Cui X., Zhang H., Zhang L., Dong Y., Zhai X. (2025). Soil Salinity Estimation Incorporating Environmental Covariables Using UAV Remote Sensing for Precision Field Management. Comput. Electron. Agric..

[45] Cui X., Han W., Zhang H., Cui J., Ma W., Zhang L., Li G. (2023). Estimating Soil Salinity under Sunflower Cover in the Hetao Irrigation District Based on Unmanned Aerial Vehicle Remote Sensing. Land Degrad. Dev..

[46] Li W., Liu C., Yang Y., Awais M., Li W., Ying P., Ru W., Cheema M.J.M. (2022). A UAV-Aided Prediction System of Soil Moisture Content Relying on Thermal Infrared Remote Sensing. Int. J. Environ. Sci. Technol..

[47] Cui X., Han W., Dong Y., Zhai X., Ma W., Zhang L., Huang S. (2023). Estimating and Mapping Soil Salinity in Multiple Vegetation Cover Periods by Using Unmanned Aerial Vehicle Remote Sensing. Remote Sens..

[48] Zhang L., Wang A., Zhang H., Zhu Q., Zhang H., Sun W., Niu Y. (2024). Estimating Leaf Chlorophyll Content of Winter Wheat from UAV Multispectral Images Using Machine Learning Algorithms under Different Species, Growth Stages, and Nitrogen Stress Conditions. Agriculture.

[49] Yan P., Feng Y., Han Q., Wu H., Hu Z., Kang S. (2025). Revolutionizing Crop Phenotyping: Enhanced UAV LiDAR Flight Parameter Optimization for Wide-Narrow Row Cultivation. Remote Sens. Environ..

[50] Awais M., Li W., Cheema M.J.M., Hussain S., Shaheen A., Aslam B., Liu C., Ali A. (2022). Assessment of Optimal Flying Height and Timing Using High-Resolution Unmanned Aerial Vehicle Images in Precision Agriculture. Int. J. Environ. Sci. Technol..

[51] Zhu W., Feng Z., Dai S., Zhang P., Wei X. (2022). Using UAV Multispectral Remote Sensing with Appropriate Spatial Resolution and Machine Learning to Monitor Wheat Scab. Agriculture.

[52] Hu G., Ren Z., Chen J., Ren N., Mao X. (2024). Using the MSFNet Model to Explore the Temporal and Spatial Evolution of Crop Planting Area and Increase Its Contribution to the Application of UAV Remote Sensing. Drones.

[53] Wang S., Han Y., Chen J., Pan Y., Cao Y., Meng H. (2020). A Transfer-Learning-Based Feature Classification Algorithm for UAV Imagery in Crop Risk Management. Desalin. Water Treat..

[54] Su J., Zhu X., Li S., Chen W.-H. (2023). AI Meets UAVs: A Survey on AI Empowered UAV Perception Systems for Precision Agriculture. Neurocomputing.

[55] Chen Z., Fang Y., Yin J., Lv S., Sheikh Muhammad F., Liu L. (2024). A Novel Lightweight YOLOv8-PSS Model for Obstacle Detection on the Path of Unmanned Agricultural Vehicles. Front. Plant Sci..

[56] Syed T.N., Zhou J., Lakhiar I.A., Marinello F., Gemechu T.T., Rottok L.T., Jiang Z. (2025). Enhancing Autonomous Orchard Navigation: A Real-Time Convolutional Neural Network-Based Obstacle Classification System for Distinguishing ‘Real’ and ‘Fake’ Obstacles in Agricultural Robotics. Agriculture.

[57] Zhou X., Chen W., Wei X. (2024). Improved Field Obstacle Detection Algorithm Based on YOLOv8. Agriculture.

[58] Lv R., Hu J., Zhang T., Chen X., Liu W. (2025). Crop-Free-Ridge Navigation Line Recognition Based on the Lightweight Structure Improvement of YOLOv8. Agriculture.

[59] Wang Q., Qin W., Liu M., Zhao J., Zhu Q., Yin Y. (2024). Semantic Segmentation Model-Based Boundary Line Recognition Method for Wheat Harvesting. Agriculture.

[60] Wang C., Chen X., Jiao Z., Song S., Ma Z. (2025). An Improved YOLOP Lane-Line Detection Utilizing Feature Shift Aggregation for Intelligent Agricultural Machinery. Agriculture.

[61] Zhang Q., Chen Q., Xu L., Xu X., Liang Z. (2023). Wheat Lodging Direction Detection for Combine Harvesters Based on Improved K-Means and Bag of Visual Words. Agronomy.

[62] Jiang Y., Li W., Liu J., Rehman M.M.U., Xie B., Wang J. (2025). Generalized Perception of Tree-Row with Distribution-Peak Feature in 3D Point Cloud for Various Orchards Navigation. Smart Agric. Technol..

[63] Xu J., Liu H., Shen Y., Yang G., Zhou H., Wang S. (2024). Point Clouds Classification and Segmentation for Nursery Trees Based on Improved PointNet plus plus Model. Chin. J. Lasers.

[64] Wen C., Wang H., Luo C., Fu W., Zhu Q., Yin Y., Meng Z. (2023). Development and Testing of a Ground Recognition System for Tractor Field Operations. Comput. Electron. Agric..

[65] Ma Z., Wang X., Chen X., Hu B., Li J. (2025). Advances in Crop Row Detection for Agricultural Robots: Methods, Performance Indicators, and Scene Adaptability. Agriculture.

[66] Ahmed S., Qiu B., Ahmad F., Kong C.-W., Xin H. (2021). A State-of-the-Art Analysis of Obstacle Avoidance Methods from the Perspective of an Agricultural Sprayer UAV’s Operation Scenario. Agronomy.

[67] Chen Z., Yin J., Farhan S.M., Liu L., Zhang D., Zhou M., Cheng J. (2026). A Comprehensive Review of Obstacle Avoidance for Autonomous Agricultural Machinery in Multi-Operational Environment. Artif. Intell. Agric..

[68] Wang Y., Zhang Z., Jia W., Ou M., Dong X., Dai S. (2025). A Review of Environmental Sensing Technologies for Targeted Spraying in Orchards. Horticulturae.

[69] Wu H., Wang X., Chen X., Zhang Y., Zhang Y. (2025). Review on Key Technologies for Autonomous Navigation in Field Agricultural Machinery. Agriculture.

[70] Yu Y., Li Z., Dai B., Pan J., Xu L. (2025). High-Precision Mapping and Real-Time Localization for Agricultural Machinery Sheds and Farm Access Roads Environments. Agriculture.

[71] Qu J., Gu Y., Qiu Z., Guo K., Zhu Q. (2025). Development of an Orchard Inspection Robot: A ROS-Based LiDAR-SLAM System with Hybrid A*-DWA Navigation. Sensors.

[72] Guan X., Ge H., Nie S., Ding Y. (2025). Research on Agricultural Autonomous Positioning and Navigation System Based on LIO-SAM and Apriltag Fusion. Agronomy.

[73] Zhu F., Chen J., Guan Z., Zhu Y., Shi H., Cheng K. (2024). Development of a Combined Harvester Navigation Control System Based on Visual Simultaneous Localization and Mapping-Inertial Guidance Fusion. J. Agric. Eng..

[74] Liu T., Zhang B., Tan Q., Zhou J., Yu S., Zhu Q., Bian Y. (2024). Immersive Human-Machine Teleoperation Framework for Precision Agriculture: Integrating UAV-Based Digital Mapping and Virtual Reality Control. Comput. Electron. Agric..

[75] Shen Y., Shen Y., Zhang Y., Huo C., Shen Z., Su W., Liu H. (2025). Research Progress on Path Planning and Tracking Control Methods for Orchard Mobile Robots in Complex Scenarios. Agriculture.

[76] Cao G., Zhang B., Li Y., Wang Z., Diao Z., Zhu Q., Liang Z. (2025). Environmental Mapping and Path Planning for Robots in Orchard Based on Traversability Analysis, Improved LeGO-LOAM and RRT* Algorithms. Comput. Electron. Agric..

[77] Jiang Q., Shen Y., Liu H., Khan Z., Sun H., Huang Y. (2025). A Hybrid Path Planning Algorithm for Orchard Robots Based on an Improved D* Lite Algorithm. Agriculture.

[78] Shi M., Feng X., Pan S., Song X., Jiang L. (2023). A Collaborative Path Planning Method for Intelligent Agricultural Machinery Based on Unmanned Aerial Vehicles. Electronics.

[79] Zhang Y., Li Y., Feng Q., Yan L., Duan H., Deng Y. (2025). Vehicle Move-Stop Motion Planning for Fruit Picking Robot with Discrete Picking Targets. Advances in Guidance, Navigation and Control.

[80] Zhao X., Lu E., Tang Z., Luo C., Xu L., Wang H. (2024). Trajectory Prediction Method for Agricultural Tracked Robots Based on Slip Parameter Estimation. Comput. Electron. Agric..

[81] Zhang Y., Shen Y., Liu H., He S., Khan Z. (2025). A Composite Sliding Mode Controller with Extended Disturbance Observer for 4WSS Agricultural Robots in Unstructured Farmlands. Comput. Electron. Agric..

[82] Zhu L., Sun W., Zhang Q., Lu E., Xue J., Sha G. (2025). Tractor Path Tracking Control Method Based on Prescribed Performance and Sliding Mode Control. Agriculture.

[83] Lu E., Xue J., Chen T., Jiang S. (2023). Robust Trajectory Tracking Control of an Autonomous Tractor-Trailer Considering Model Parameter Uncertainties and Disturbances. Agriculture.

[84] Ding C., Ding S., Wei X., Mei K. (2022). Composite SOSM Controller for Path Tracking Control of Agricultural Tractors Subject to Wheel Slip. ISA Trans..

[85] Ding C., Ding S., Wei X., Mei K. (2022). Output Feedback Sliding Mode Control for Path-Tracking of Autonomous Agricultural Vehicles. Nonlinear Dyn..

[86] Sun J., Li Q., Ding S., Xing G., Chen L. (2023). Fixed-Time Generalized Super-Twisting Control for Path Tracking of Autonomous Agricultural Vehicles Considering Wheel Slipping. Comput. Electron. Agric..

[87] Yang W., Ding S., Ding C. (2024). Fast Supertwisting Sliding Mode Control with Antipeaking Extended State Observer for Path-Tracking of Unmanned Agricultural Vehicles. IEEE Trans. Ind. Electron..

[88] Ji X., Ding S., Cui B., Ding C., Wei X. (2023). Barrier Function-Based Nonsingular Terminal Sliding Mode Control for Path Tracking of Tractor-like with Experimental Validation. IEEE Trans. Circuits Syst. II Express Briefs.

[89] Ji X., Ding S., Wei X., Cui B. (2023). Path Tracking of Unmanned Agricultural Tractors Based on a Novel Adaptive Second-Order Sliding Mode Control. J. Frankl. Inst..

[90] Chen Z., Yin J., Farhan S.M., Lin Z., Zhou M. (2025). Adaptive Path Tracking Control of Unmanned Agricultural Machinery with Fixed-Time Super-Twisting Sliding Mode Based on RLS-ELM. Front. Plant Sci..

[91] Li J., Shang Z., Li R., Cui B. (2022). Adaptive Sliding Mode Path Tracking Control of Unmanned Rice Transplanter. Agriculture.

[92] Chen W., Xu T., Liu J., Wang M., Zhao D. (2019). Picking Robot Visual Servo Control Based on Modified Fuzzy Neural Network Sliding Mode Algorithms. Electronics.

[93] Wang A., Ji X., Song Q., Wei X., Chen W., Wang K. (2025). Path Tracking Controller and System Design for Agricultural Tractors Based on Improved Stanley and Sliding Mode Algorithms Considering Sideslip Compensation. Agronomy.

[94] Sun Y., Cui B., Ji F., Wei X., Zhu Y. (2022). The Full-Field Path Tracking of Agricultural Machinery Based on PSO-Enhanced Fuzzy Stanley Model. Appl. Sci..

[95] Wang X., Zhang B., Du X., Chen H., Zhu T., Wu C. (2025). Self-Adjusting Look-Ahead Distance of Precision Path Tracking for High-Clearance Sprayers in Field Navigation. Agronomy.

[96] Wang X., Zhang B., Du X., Hu X., Wu C., Cai J. (2025). An Adaptive Path Tracking Controller with Dynamic Look-Ahead Distance Optimization for Crawler Orchard Sprayers. Actuators.

[97] Jia W., Zhang Z., Dong X., Ou M., Gao R., Wang Y., Yang Q., Wang X. (2025). Quantification and Optimization of Straight-Line Attitude Control for Orchard Weeding Robots Using Adaptive Pure Pursuit. Agriculture.

[98] Jia W., Tian Y., Duan H., Luo R., Lian J., Ruan C., Zhao D., Li C. (2020). Autonomous Navigation Control Based on Improved Adaptive Filtering for Agricultural Robot. Int. J. Adv. Robot. Syst..

[99] Lu E., Ma Z., Li Y., Xu L., Tang Z. (2020). Adaptive Backstepping Control of Tracked Robot Running Trajectory Based on Real-Time Slip Parameter Estimation. Int. J. Agric. Biol. Eng..

[100] Wang A., Wang Y., Ji X., Wang K., Qian M., Wei X., Song Q., Chen W., Zhang S. (2024). Fuzzy Backstepping Controller for Agricultural Tractor-Trailer Vehicles Path Tracking Control with Experimental Validation. Front. Plant Sci..

[101] Zhang S., Wei X., Liu C., Ge J., Cui X., Wang F., Wang A., Chen W. (2025). Adaptive Path Tracking and Control System for Unmanned Crawler Harvesters in Paddy Fields. Comput. Electron. Agric..

[102] Zhang Y., Chen W.-H., Liu H., Wang H., Khan Z., Shen Y. (2025). High-Order Disturbance Observer Enhanced Explicit Nonlinear MPC for Robust Trajectory Tracking of Agricultural Robots. IEEE Trans. Ind. Electron..

[103] Jia H., Chen W., Su Z., Sun Y., Qian Z., Huang L. (2025). AI-Driven Cooperative Control for Autonomous Tractors and Implements: A Comprehensive Review. AgriEngineering.

[104] Wang Y., Zhu X., Yang D., Xu L., Guo P., Chen W.-H. (2025). High Efficient Cooperation Control of Distributed Motor Drive Electric Tractor Considering Complex Farmland Operation Conditions. IEEE Trans. Transp. Electrif..

[105] Lu E., Zhao X., Ma Z., Xu L., Liu Y. (2024). Robust Leader-Follower Control for Cooperative Harvesting Operation of a Tractor-Trailer and a Combine Harvester Considering Confined Space. IEEE Trans. Intell. Transp. Syst..

[106] Li Y., Xu L., Lv L., Shi Y., Yu X. (2022). Study on Modeling Method of a Multi-Parameter Control System for Threshing and Cleaning Devices in the Grain Combine Harvester. Agriculture.

[107] Yang L., Yaoming L., Liya L., Li Zhang X., Maolin S. (2024). Modeling and Control Methods of a Multi-Parameter System for Threshing and Cleaning in Grain Combine Harvesters. Comput. Electron. Agric..

[108] Qian Y., Quan L., Diao J., Xu L., Xiang Z., Zhu X. (2025). Research on Speed Control of an Electrically Driven Threshing Cylinder in a Combine Harvester Based on Inertia Parameter Identification. INMATEH Agric. Eng..

[109] Wang Q., Zhao J., Meng Z., Qin W., Wang F., Zhao C., Zhu Q., Wen C., Yin Y. (2025). A Fuzzy Decision-Making Algorithm-Based Header Height Measurement System for Combine Harvester. Measurement.

[110] Ruan M., Jiang H., Zhou H., Ye J., Hu J. (2022). Design and Test of Automatic Control System for Header Height of Combine Harvester. INMATEH Agric. Eng..

[111] Wang Q., Meng Z., Wen C., Qin W., Wang F., Zhang A., Zhao C., Yin Y. (2024). Grain Combine Harvester Header Profiling Control System Development and Testing. Comput. Electron. Agric..

[112] Chen S., Qi J., Gao J., Chen W., Fei J., Meng H., Ma Z. (2025). Research on the Control System for the Conveying and Separation Experimental Platform of Tiger Nut Harvester Based on Sensing Technology and Control Algorithms. Agriculture.

[113] Chen J., Ji J., Ji K., Chen Y. (2025). Deep Learning-Driven Predictive Control Method for Optimizing Combine Harvester Operation Speed. Eng. Agríc..

[114] Sun Y., Luo Y., Chai X., Zhang P., Zhang Q., Xu L., Wei L. (2021). Double-Threshold Segmentation of Panicle and Clustering Adaptive Density Estimation for Mature Rice Plants Based on 3D Point Cloud. Electronics.

[115] Li Z., Wang R., Ding R. (2025). A Review of Crop Attribute Monitoring Technologies for General Agricultural Scenarios. AgriEngineering.

[116] Cui Z., Liu P., Hu J., Guo E., He Z., Chai X., Chen H., Song Z., Xu L. (2026). An Online Precision Estimation Method for Impurity Mass in Grain Flow of Rice Combine Harvesters Based on Multimodal Data Fusion. Comput. Electron. Agric..

[117] Chen J., Shi T., Li Y., Zhu Y., Niu C. (2025). A Method for Monitoring Rice Seed Loss Based on WOA-BP Algorithm. Eng. Agríc..

[118] Qian P., Lu T., Shen C., Chen S. (2021). Influence of Vibration on the Grain Flow Sensor during the Harvest and the Difference Elimination Method. Int. J. Agric. Biol. Eng..

[119] Ma Z., Liu C., Zhang J., Wang S., Li Y. (2025). Study on Blockage Monitoring of Differential-Speed Roller Potato-Soil Separation Device. Comput. Electron. Agric..

[120] Jia W., Zhang Y., Lian J., Zheng Y., Zhao D., Li C. (2020). Apple Harvesting Robot under Information Technology: A Review. Int. J. Adv. Robot. Syst..

[121] Li T., Xie F., Zhao Z., Zhao H., Guo X., Feng Q. (2023). A Multi-Arm Robot System for Efficient Apple Harvesting: Perception, Task Plan and Control. Comput. Electron. Agric..

[122] Jiang Y., Zhao S., Liu J., Wu S., Jiang Y., Jin Y. (2025). Review of Dual-Arm Parallel and Collaborative Motion: Methods, Progress and Applications in Agriculture. Comput. Electron. Agric..

[123] Yuan F., Wang X., Shi Y., Wang L., Zhang X., Lu W., Zheng E. (2025). Prediction of the Viscoelastic Parameters of Asparagus and Optimization of the Harvesting Robot Gripping Force Based on a Back-Propagation Neural Network. Smart Agric. Technol..

[124] Elsherbiny O., Gao J., Ma M., Qureshi W.A., Mosha A.H. (2025). Developing an IoT-Driven Delta Robot to Stimulate the Growth of Mulberry Branch Cuttings Cultivated Aeroponically Using Machine Vision Technology. Comput. Electron. Agric..

[125] Xu L., Xie Y., Chen X., Chen Y., Kang Z., Huang P., Zou Z., He Y., Yang N., Peng Y. (2022). Design of an Efficient Combined Multipoint Picking Scheme for Tea Buds. Front. Plant Sci..

[126] Gu X., Tang Z., Wang B. (2025). Sensor-Centric Intelligent Systems for Soybean Harvest Mechanization in Challenging Agro-Environments of China: A Review. Sensors.

[127] Jiang L., Wang G., Xu B., Husnain N., Wang Q. (2025). Intelligent Systems for Combine Harvesters: A Comprehensive Review of Technologies and Trends. IEEE Access.

[128] Guan X., Shi L., Ge H., Ding Y., Nie S. (2025). Development, Design, and Improvement of an Intelligent Harvesting System for Aquatic Vegetable *Brasenia schreberi*. Agronomy.

[129] Fang H., Li J., Zhang Q., Cheng G., Lu J., Zhang J. (2025). Design and Experiment for a Crawler Self-Propelled Potato Combine Harvester for Hilly and Mountainous Areas. Agriculture.

[130] Tang Z., Wang H., Liu S., Lu D., Tang Y. (2023). Development of Structure and Control System of Self-Propelled Small Green Vegetables Combine Harvester. J. Agric. Sci. Technol..

[131] Liu H., Du Z., Shen Y., Du W., Zhang X. (2024). Development and Evaluation of an Intelligent Multivariable Spraying Robot for Orchards and Nurseries. Comput. Electron. Agric..

[132] Zhu C., Hao S., Liu C., Wang Y., Jia X., Xu J., Guo S., Huo J., Wang W. (2024). An Efficient Computer Vision-Based Dual-Face Target Precision Variable Spraying Robotic System for Foliar Fertilisers. Agronomy.

[133] Gao X., Gao J., Qureshi W.A. (2025). Applications, Trends, and Challenges of Precision Weed Control Technologies Based on Deep Learning and Machine Vision. Agronomy.

[134] Zheng S., Zhao X., Fu H., Tan H., Zhai C., Chen L. (2025). Design and Experimental Evaluation of a Smart Intra-Row Weed Control System for Open-Field Cabbage. Agronomy.

[135] Liu J., Zhao S., Li N., Faheem M., Zhou T., Cai W., Zhao M., Zhu X., Li P. (2019). Development and Field Test of an Autonomous Strawberry Plug Seeding Transplanter for Use in Elevated Cultivation. Appl. Eng. Agric..

[136] Zhong W., Yang W., Wang Y., Dong X., Wang X., Jia W., Ou M. (2025). Intelligent Pesticide Application System for Intercropping: Development, Challenges, and Solutions Based on Monoculture Machinery. Cogent Food Agric..

[137] Tang S., Yan Z., Xue Y., Lim K.M., Zhu Y. (2025). Deep Transferable Model with Multi-Head Attention for Small Sample Fault Diagnosis of Hydraulic Pumps. Eng. Appl. Artif. Intell..

[138] Tang S., Jiang Y., Zhou T., Zhu Y., Lim K.M. (2025). Bayesian Algorithm Optimized Deep Model for Multi-Signal Fault Identification of Hydraulic Pumps. Alex. Eng. J..

[139] Li X., Li M., Liu B., Lv S., Liu C. (2024). A Novel Transformer Network Based on Cross-Spatial Learning and Deformable Attention for Composite Fault Diagnosis of Agricultural Machinery Bearings. Agriculture.

[140] Chakroun A., Hani Y., Elmhamedi A., Masmoudi F. (2024). A Predictive Maintenance Model for Health Assessment of an Assembly Robot Based on Machine Learning in the Context of Smart Plant. J. Intell. Manuf..

[141] Bai X., Chen Q., Song X., Hong W. (2025). Advancing Agricultural Machinery Maintenance: Deep Learning-Enabled Motor Fault Diagnosis. IEEE Access.

[142] Wang H., Lao L., Zhang H., Tang Z., Qian P., He Q. (2025). Structural Fault Detection and Diagnosis for Combine Harvesters: A Critical Review. Sensors.

[143] Tang Z., Wang H., Tian L., Lao L., Sun H. (2025). Enhancing Grain Harvester Fatigue Reliability to Support Sustainable Agriculture: A Review of Research Status and Prospects. Sci. Prog..

[144] Zhang C., Song J., Yin X., Cai J., Liang Y., Lu J. (2025). Digital Twin-Based Approaches for Agricultural Machinery Damage Prediction and Maintenance: A Review. J. Comput. Des. Eng..

[145] Zhang S., Zang C., Yang Z., Tang L., Wang K., Wang A., Chen W., Song Q., Wei X. (2025). Research on Fault Prediction and Speed Control System for Unmanned Combine Harvesters Based on IPSO-SVM and Fuzzy Logic. Front. Plant Sci..

[146] Weng S., Yuan C., He Y., Shen J., Chen L., Xu L., Zhu Z., Yu Q., Sun Z. (2024). Neural Network Energy Management Strategy for Plug-in Hybrid Electric Combine Harvesters Based on Quasi-Periodic Samples. Eng. Appl. Artif. Intell..

[147] Sia W.R., Saufi M.S.R.M., Isham M.F.B., Leong M.S. (2025). One-Shot Transfer Learning with Limited Data Sample for Bearing Component Fault Diagnosis. Int. J. Hydromechatron..

[148] Tang S., Jiang Y., Su H., Lim K.M., Zheng Z., Zhu Y. (2025). Wear Defect Detection of Hydraulic Pump Using a Hybrid Method of VGG and LSTM. Int. J. Hydromechatron..

[149] Ding R., Sun G., Zhang J., Peng L., Cheng M., Xu B., Yang H. (2025). A Review of Independent Metering Control System for Mobile Machinery. Int. J. Hydromechatron..

[150] Bai X., Yue J., Guan R., Cui Z., Li R. (2025). A Closed-Loop Evaluation Identification Algorithm for Rubber Mixing Process Based on Physical Information and Deep Learning. Int. J. Hydromechatron..

[151] Zhang X., Liao H., Xu Y. (2026). Detecting Fruit Ripeness in the Wild: A Robust Deep Learning Model and the Comprehensive and Diverse Fruit Ripeness Benchmark. Eng. Appl. Artif. Intell..

[152] Lv P., Xue J., Wei W., Liu S., Gao W., Sun H., Miao H., Wang W. (2026). An Advanced Detector and Dual-Shortest Distance Intersection Algorithm for Navigation Path Extraction in Complex Orchards. Eng. Appl. Artif. Intell..

[153] Taha M.F., Mao H., Zhang Z., Elmasry G., Awad M.A., Abdalla A., Mousa S., Elwakeel A.E., Elsherbiny O. (2025). Emerging Technologies for Precision Crop Management towards Agriculture 5.0: A Comprehensive Overview. Agriculture.

[154] Gong R., Zhang H., Li G., He J. (2025). Edge Computing-Enabled Smart Agriculture: Technical Architectures, Practical Evolution, and Bottleneck Breakthroughs. Sensors.

[155] Yu P., Teng F., Zhu W., Shen C., Chen Z., Song J. (2025). Cloud-Edge-Device Collaborative Computing in Smart Agriculture: Architectures, Applications, and Future Perspectives. Front. Plant Sci..

[156] Chen H., Li H., Zhao J., Ruan C., Huang L. (2025). Enhancing Crop Disease Recognition via Prompt Learning-Based Progressive Mixup and Contrastive Language-Image Pre-Training Dynamic Calibration. Eng. Appl. Artif. Intell..

[157] Xiang J., Wang L., Li L., Lai K.-H., Cai W. (2024). Classification-Design-Optimization Integrated Picking Robots: A Review. J. Intell. Manuf..

[158] Wang B., Du X., Wang Y., Mao H. (2024). Multi-Machine Collaboration Realization Conditions and Precise and Efficient Production Mode of Intelligent Agricultural Machinery. Int. J. Agric. Biol. Eng..

[159] Wadhwa D., Malik K. (2026). Deep Learning Generalized Hybrid Models for Multi-Species Crop Disease Classification with Explainable Insights. Eng. Appl. Artif. Intell..

[160] Zhang R., Zhu H., Chang Q., Mao Q. (2025). A Comprehensive Review of Digital Twins Technology in Agriculture. Agriculture.

